# EMILIN1 emerges as a TGFβ/SETDB1-regulated secreted biomarker in Duchenne muscular dystrophy

**DOI:** 10.1038/s41419-026-08825-8

**Published:** 2026-05-09

**Authors:** Maeva Zamperoni, Laura Muraine, Minh-Y Tran, Alice Granados, Anne Bigot, Valentin Petit, Mona Bensalah, Jessica Ohana, Véronique Legros, Ekaterina Boyarchuk, Johanna Bruce, Guillaume Chevreux, Véronique Joliot, Elisa Negroni, Maryline Moulin, Capucine Trollet, Slimane Ait-Si-Ali

**Affiliations:** 1 Université Paris Cité, CNRS, Epigenetics and Cell Fate, UMR7216, F-75013 Paris, France; 2 Sorbonne Université, Inserm, Institut de Myologie, Centre de Recherche En Myologie, F-75013 Paris, France; 3 Université Paris Cité, CNRS, Institut Jacques Monod, UMR7592, F-75013 Paris, France; 4 Proteom’IC facility, Université Paris Cité, CNRS, INSERM, Institut Cochin, F-75014 Paris, France

**Keywords:** Cell signalling, Epigenetics, Proteomics

## Abstract

Duchenne muscular dystrophy (DMD) is an incurable muscle-wasting disorder characterized by chronic membrane damage, inflammation, and progressive fibrosis. Fibrosis in DMD is driven by sustained TGFβ signaling, which promotes extracellular matrix (ECM) accumulation. We previously showed that SETDB1 sustains the TGFβ-induced fibrotic response in DMD myotubes. Here, we further show that SETDB1 modulates the TGFβ-induced secretome, particularly by regulating ECM-related proteins. Comparison of the basal secretome from DMD patient-derived myotubes and healthy controls revealed a distinct disease-specific profile. Integrating both secretome analyses, we identified EMILIN1, an ECM glycoprotein not previously studied in skeletal muscle, as a robust shared candidate; EMILIN1 is enriched in the DMD secretome, further upregulated by TGFβ, and downregulated upon SETDB1 depletion. We confirmed EMILIN1 overexpression in DMD patient muscle biopsies, validating its pathological relevance. Functionally, *EMILIN1* depletion modulated myogenic differentiation and reduced expression of the fibrotic marker *SERPINE1*. These findings establish EMILIN1 as a novel secreted regulator of myogenesis and fibrosis, and implicate SETDB1 in shaping the TGFβ-dependent secretome in DMD. Our integrative proteomic approach provides new insights into the molecular drivers of impaired regeneration in DMD and highlights potential therapeutic targets.

## Introduction

Skeletal muscle is a highly organized structure composed of various tissues, including blood and lymphatic vessels, contractile muscle fibers, and connective tissue [1]. The cooperation between these tissues guarantees the regenerative ability of skeletal muscle upon injury. Normally, after mechanical trauma, exposure to toxins or infections, skeletal muscle retains the ability to completely repair, thanks to a sequential and well-orchestrated series of events, referred to as muscle regeneration. However, in conditions of disease, like in DMD, the inflammatory response becomes chronic, leading to fibrosis, defined as an excessive accumulation of ECM components, and to fat accumulation, which ultimately replaces functional muscle tissue with non-functional one [2, 3].

DMD is an X-chromosome-linked recessive muscle wasting disorder with a prevalence of approximately 1 in 5000 live male births [4]. DMD is due to deleterious mutations in the *DMD* gene leading to the lack of a functional Dystrophin protein, and thus to a significant fragility of the sarcolemma. This weakening of the sarcolemma contributes to repeated cycles of muscle fiber injury and repair, ultimately resulting in progressive muscle degeneration. Over time, this degeneration leads to extensive muscle wasting [5, 6]. One of the key pathological features accompanying this process is fibrosis, which is sustained by the TGFβ pathway, overactivated in DMD [7].

We recently identified the lysine methyltransferase and epigenetic regulator SETDB1 as a modulator of TGFβ signaling in DMD [8]. SETDB1 catalyzes histone H3 lysine 9 tri-methylation (H3K9me3), an epigenetic modification primarily associated with heterochromatin formation and gene repression, thereby contributing to the regulation of the cells’ functional states (e.g., stemness, proliferation, differentiation). In proliferating myoblasts, SETDB1 remains predominantly nuclear, where it prevents the expression of late differentiation genes through H3K9me3. Upon Wnt signaling activation at the onset of terminal differentiation, SETDB1 is exported to the cytoplasm, facilitating myotube formation [9]. TGFβ induces nuclear accumulation of SETDB1 in healthy myotubes, while SETDB1 is constantly accumulated in DMD myotube nuclei with intrinsic overactivated TGFβ pathway [8]. Moreover, our previous results showed that SETDB1 regulates the expression of TGFβ-dependent secreted factors impacting fibrosis and myogenic differentiation. We demonstrated that the conditioned medium (CM) of TGFβ-treated myotubes impairs myoblasts differentiation and increases the expression of pro-fibrotic genes [8].

Here, we investigated how SETDB1 modulates the secretome of DMD myotubes using proteomics, with a focus on TGFβ-dependent targets. In parallel, we compared the secretome from DMD *versus* control myotubes under basal conditions to identify deregulated secreted factors associated with the disease context. Together, these complementary approaches allowed us to dissect both basal and TGFβ-induced alterations in the DMD secretome. We found that SETDB1 modulates the TGFβ-induced secretome, particularly by regulating the expression of secreted and ECM-related proteins, such as EMILIN1 (Elastin Microfibril Interface (EMI) Located proteIN 1). EMILIN1 is a glycoprotein primarily expressed in the ECM, where it contributes to various cellular processes through its C-terminal gC1q domain, which mediates interactions with integrins, promoting cell adhesion and migration, and through its EMI domain, involved in TGFβ processing [10]. Studies performed in different contexts, like blood vessels [11], skin psoriasis [12] and cancer [13] revealed an inhibitory role of EMILIN1 on TGFβ signaling.

The role and function of EMILIN1 in skeletal muscle have not been described yet. Here, we show that EMILIN1 is more highly expressed in DMD muscle cells and patient biopsies compared to healthy controls and may play a key role in regulating myogenic differentiation. Loss of EMILIN1 in myoblasts and myotubes results in increased expression of early myogenic markers, while late markers are reduced. Additionally, EMILIN1 modulates the expression of the fibrotic marker SERPINE1, which is regulated by both TGFβ and SETDB1. Notably, EMILIN1 depletion leads to a decrease in SERPINE1 expression. Our findings suggest that EMILIN1 is a TGFβ- and SETDB1-dependent secreted factor involved in regulating proper muscle cell differentiation and fibrotic response.

## Results

### Proteomic profiling reveals many secreted factors regulated by TGFβ and SETDB1 in DMD myotubes

Using human immortalized myoblasts derived from healthy individuals or DMD patients [14], we have previously shown that CM from TGFβ-treated myotubes impairs the receiving myoblasts differentiation and promotes the expression of pro-fibrotic genes [8]. Interestingly, SETDB1 knockdown (KD) in the CM-producing myotubes partially counteracts this effect [8].

**Fig. 1:**
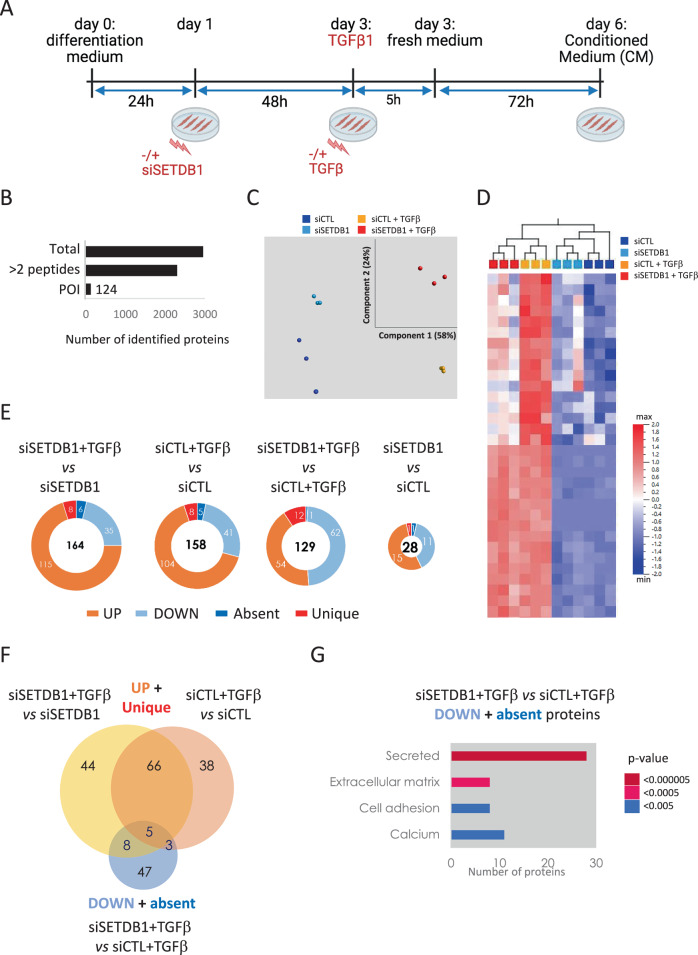
Proteomic profiling of the TGFβ-responsive and SETDB1-dependent secretome in DMD myotubes. **A** Diagram of the secretome experiment. **B** Secretome analysis of the mass spectrometry results on the condition media described in **A** (*N* = 3, DMD #1), the complete list of identified proteins is shown in Table S1. Proteins of interest (POI) from the ANOVA analysis with *p* < 0.001. **C** Principal component analysis (PCA) of the significant target from the ANOVA analysis. The two axes represent the first and second principal components, defined as the directions that maximize the variance of the projected data from the secretome analysis across the four conditions: siCTL, siCTL+TGFβ, siSETDB1, and siSETDB1+TGFβ. The percentage indicated for each axis corresponds to the proportion of the total variance captured by that component. Component 1 separates samples based on TGFβ treatment, whereas Component 2 clusters samples according to siSETDB1 or siCTL treatment. **D** Selected area of the heatmap (ANOVA. *p* < 0.001) showing the candidates that are upregulated upon TGFβ treatment (yellow) compared to basal condition (blue) and that decrease in siSETDB1+TGFβ (red). Complete heatmap is shown in Fig S1A. **E** Doughnut plot showing the number of proteins from secretome analysis that are altered (down-regulated, up-regulated, absent or uniquely found). Total number of significant proteins is also indicated (T-test. *p* < 0.01). A complete list of altered proteins along with the fold changes is shown in Tables S2–S5. **F** Venn diagram of the shared proteins between the two-by-two comparison from (**E**). The Venn Diagram shows the intersection between up+unique proteins from comparison with siSETDB1+TGFβ vs siSETDB1 (impact of TGFβ in the SETDB1 KD) and with siCTL+TGFβ vs siCTL (impact of TGFβ) with the proteins down regulated and absent in siSETDB1+TGFβ vs siCTL+TGFβ (impact of SETDB1 KD on TGFβ treatment). **G** David bioinformatics functional annotation chart (Sherman et al.) using uniprotKB keywords (BIOLOGICAL_PROCESS, CELLULAR_COMPONENT, MOLECULAR_FUNCTION and LIGAND) using down and absent protein from siSETDB1+TGFβ versus siCTL+TGFβ comparison. Color intensity reflects enrichment significance after multiple testing correction using the Benjamini–Hochberg false discovery rate as implemented in DAVID Bioinformatics.

To identify the secreted factors mediating the inhibitory effect of the TGFβ-treated myotubes on the myoblast differentiation, we performed mass spectrometry (MS) analysis of the CM. Specifically, we analyzed CM collected from DMD myotubes upon SETDB1 KD and/or TGFβ treatment (Fig. 1A). A total of almost 3000 proteins were identified across all samples (Figs. 1B, S1A, and Table S1). The Principal Component Analysis (PCA) showed clear clustering of the samples, with the main separation driven by TGFβ treatment and SETDB1 KD (Fig. 1C).

Overall, we found a significant number of differentially enriched proteins in response to TGFβ treatment and/or SETDB1 KD (Figs. 1D, E, S1A). We reasoned that relevant candidates would be upregulated in response to TGFβ, reflecting their potential to promote fibrosis and impair differentiation. Conversely, these factors should be downregulated upon SETDB1 KD, a condition that partially attenuates the pathological effects of TGFβ [8]. Therefore, we focused on the proteins upregulated upon TGFβ treatment and decreased with SETDB1 KD (Fig. 1D).

The comparison between TGFβ-treated condition with and without SETDB1 KD (siSETDB1-TGFβ vs siCTL+TGFβ) revealed a nearly equal number of downregulated (62 + 1) and upregulated (54 + 12) proteins (Fig. 1E). The complete list of differentially detected proteins is shown in Tables S2, S5. The signature of up-regulated and unique proteins induced by TGFβ overlapped by nearly 60% between the control and SETDB1 KD conditions (Fig. 1F). Activation of the TGFβ pathway, independently of SETDB1 KD, led to an increase of secreted proteins related to the ECM (Fig. S1B, C). The promising candidates which were downregulated upon SETDB1 KD in the presence of TGFβ belong to the secreted and ECM-related protein categories (Fig. 1G). For instance, the ECM remodeling enzymes MMP14 and ADAMTS4 were upregulated in response to TGFβ, and downregulated upon SETDB1 KD (Fig. S2A). A similar pattern was observed for the ECM component COL1A2 and the immune-related factor IL11 (Fig. S2A). Regulation of MMP14 and IL11 was further confirmed at the transcript level in myotubes producing the CM, although the reduction in transcript levels induced by SETDB1 KD was less pronounced than the corresponding decrease observed at the protein level (Fig. S2B).

Together, these findings reveal many TGFβ-responsive, SETDB1-dependent secreted factors in myotubes that may ultimately impact myoblast differentiation and the fibrotic response.

### Comparative proteomic reveals differences between the basal secretome of human DMD and healthy myotubes

**Fig. 2:**
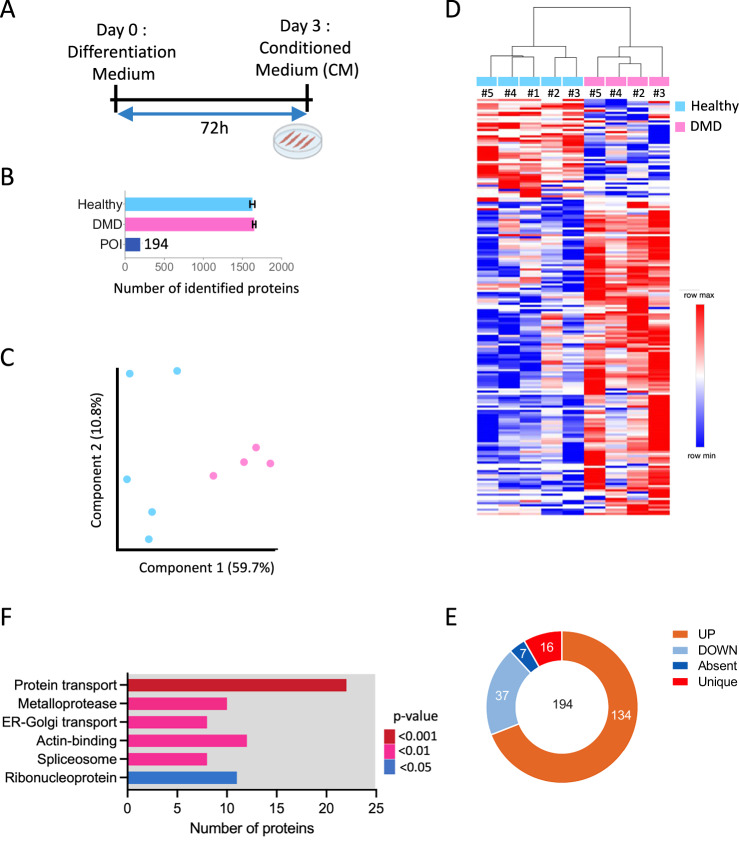
Proteomic profiling of basal secretome of DMD myotubes compared to healthy myotubes. **A** Diagram of the secretome experiment. **B** Secretome analysis of the mass spectrometry results on the conditioned media described in **A** (*N* = 5 control and *N* = 4 DMD). POI: proteins of interest. **C** PCA of the significant target from the ANOVA analysis. **D** Heatmap showing the POI (*p* < 0.05 and FC > 1.2) differentially expressed in DMD samples (pink) compared to healthy controls (blue). Detailed heat map is shown in Fig S2. **E** Doughnut plot showing the number of proteins differentially expressed in DMD samples compared to controls. **F** David bioinformatics functional annotation chart (Sherman et al.) using uniprotKB keywords (BIOLOGICAL_PROCESS, CELLULAR_COMPONENT, MOLECULAR_FUNCTION and LIGAND) using all proteins of interest from the DMD secretome data. Color intensity reflects enrichment significance after multiple testing correction using the Benjamini–Hochberg false discovery rate as implemented in DAVID Bioinformatics.

After identifying deregulated secreted proteins upon stimulation, we next examined the basal secretome of DMD muscle cells to uncover intrinsic differences from healthy controls, independent of exogenous triggers. For this we performed a comparative proteomic analysis of the CM collected from human DMD and healthy immortalized myotubes under basal conditions after 3 days of differentiation (Fig. 2A). We identified a total of approximately 1600 secreted proteins across all samples (Fig. 2B, complete heatmap shown in Fig. S3). PCA revealed a clear separation between DMD and healthy samples (Fig. 2C), indicating a distinct secretome signature associated with the disease state independently of the mutation in the *DMD* gene. Comparative secretome analysis (Fig. 2D, E) revealed that DMD myotubes are enriched in proteins involved in RNA processing (HNRNPR, SYNCRIP), vesicular trafficking (RAB11B, SEC23A), metabolic enzymes (PGK1, PKM), and cytoskeletal regulation (ARPC1A, CDC42), while structural ECM components (LAMA5) and signaling modulators (TGFβ2, ADAM10) are depleted. Functional annotation analysis using UniProtKB keywords identified enrichment in categories related to protein transport, ER–Golgi transport, metalloproteases, actin-binding, and spliceosome-associated proteins (Fig. 2F).

Together, these results indicate a shift in the DMD secretome marked by enrichment of metabolic, RNA-binding, and cytoskeletal proteins, loss of structural ECM components and TGFβ signaling modulators, and the emergence of a DMD-specific secretory signature that may contribute to impaired myofiber stability and pathological remodeling of the muscle microenvironment.

### SETDB1 depletion reveals EMILIN1 as a key TGFβ-responsive ECM protein in DMD myotubes

**Fig. 3:**
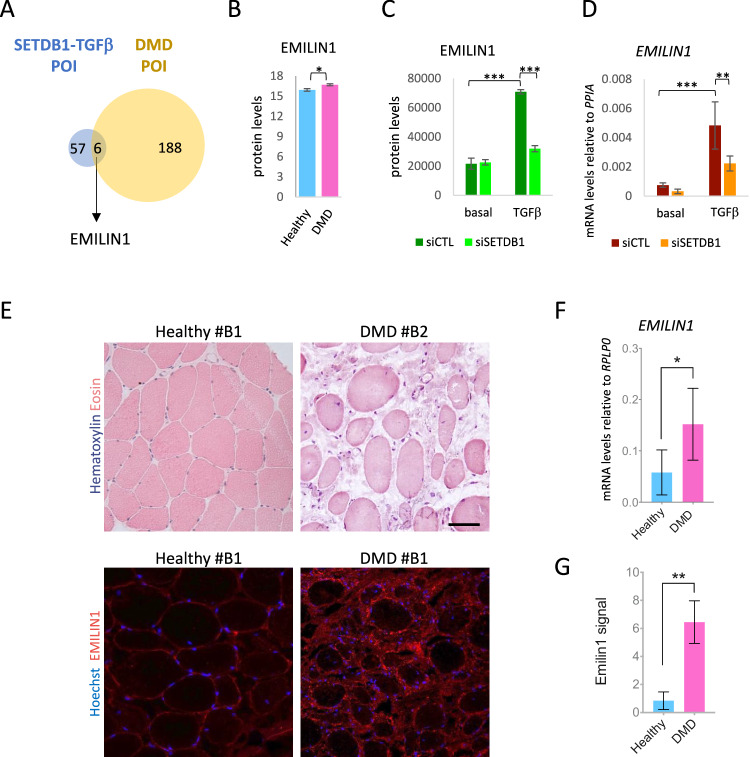
EMILIN1 is a shared secreted biomarker of the basal DMD and TGFβ–SETDB1 secretomes and is upregulated in patient muscle. **A** Venn Diagram of the shared proteins between the downregulated proteins upon siSETDB1+TGFβ (same as Fig. 1 G) and the POI of DMD secretome (same as Fig. 2E). **B** MS data analysis of EMILIN1 between healthy and DMD secretome. Data are represented as average **±** SEM. **C** MS data analysis of EMILIN1 in DMD with siSETDB1 **±** TGFβ. Data are represented as average **±**SEM. **D** RTqPCR of *EMILIN1* (*N* = 3, DMD #1). Data are represented as average **±** SEM. **E** Hematoxylin and eosin coloration of healthy and DMD muscle biopsies and immunostaining of EMILIN1 (red) and Hoechst (blue) scale bar = 50 µm. **F** RTqPCR quantification of *EMILIN1* on human muscle biopsies of healthy and DMD patients (biological replicates: *N* = 7 Healthy, *N* = 6 DMD). Data are represented as mean **±** SD. **G** Quantification of the percentage of EMILIN1 signal on healthy and DMD muscles (biological replicates *N* = 3 Healthy, *N* = 4 DMD). For all panels, data are represented with *p < 0,05, **p < 0.01; ***p < 0.001 unpaired Student’s *t* test.

In order to narrow down the list of altered proteins, we focused on the ones that are increased in DMD basal condition and also upregulated by TGFβ treatment but reduced with SETDB1 KD, by selecting proteins common to the DMD secretome and those downregulated (or absent) in the comparison siSETDB1+TGFβ *vs* siCTL+TGFβ. Our stringent analysis revealed only six candidates (EMILIN1, ARCN, NEXN, ADAM10, SSC5D, and NES), among them only EMILIN1 is upregulated in basal DMD condition, upregulated by TGFβ treatment and reduced with SETDB1 KD (Fig. 3A–C, Fig. S4A, B). As a key ECM glycoprotein, EMILIN1 is involved in structural and regulatory functions. Indeed, EMILIN1 promotes cell adhesion and migration, and it acts as a negative regulator of TGFβ by blocking its maturation and activation [10]. EMILIN1 protein level changes were confirmed also at the mRNA level, showing that DMD myotubes were indeed producing higher amounts of EMILIN1 upon TGFβ treatment and that SETDB1 KD partially reversed this effect (Fig. 3D). DMD biopsies present high levels of fibrosis and variations in fiber size (Fig. 3E) Interestingly, EMILIN1 was also found enriched, both at mRNA and protein levels in muscle tissue sections from DMD patients compared to healthy controls and localized around muscle fibers (Fig. 3E, G, Fig. S4C).

Together, these findings identify EMILIN1 as a TGFβ and SETDB1-dependent target which is enriched in the DMD basal secretome and further upregulated in response to TGFβ stimulation. Notably, EMILIN1 overexpression in muscle biopsies from DMD patients supports its pathological relevance in vivo.

### EMILIN1 plays a role in myogenic differentiation of healthy and DMD myotubes

**Fig. 4:**
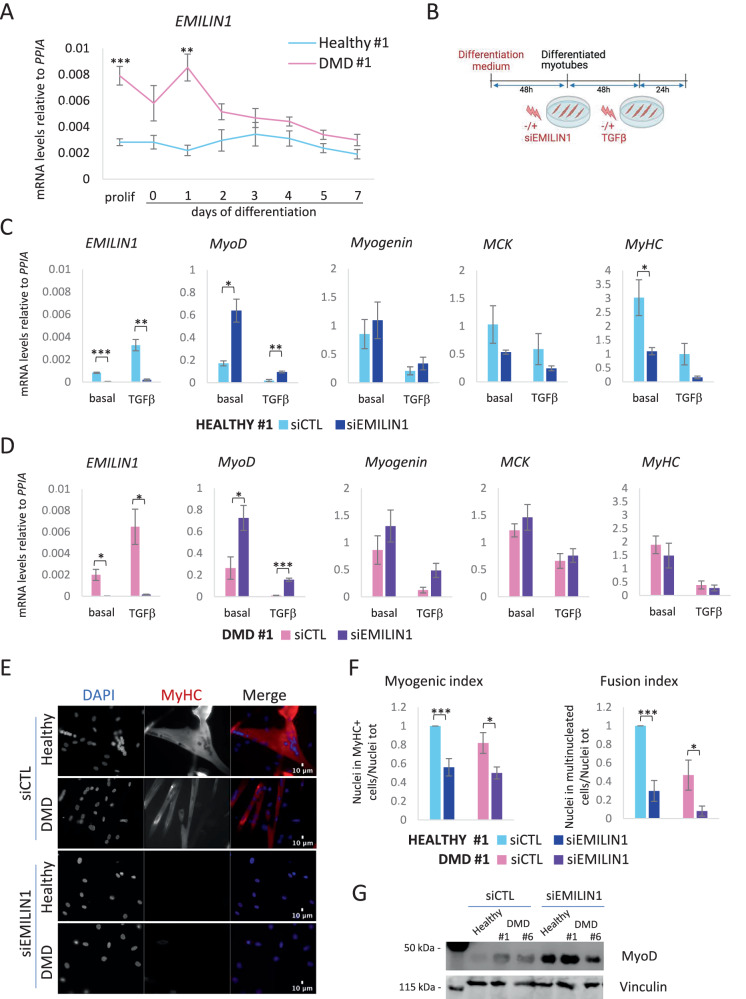
EMILIN1 KD increases the expression of early myogenic markers and inhibits late differentiation in healthy and DMD myotubes. **A** RTqPCR of *EMILIN1* in healthy and DMD proliferating myoblasts and differentiated myotubes (*N* = 4, DMD#1). **B** Diagram of experimental design. Cells were transfected with siRNAs scrambled (siCTL) or against EMILIN1 (siEMILIN1) after 2 days of differentiation (myotubes) and 2 days later treated or not with TGFβ at 20 ng/mL for 24 h. **C** RTqPCR of *EMILIN1* and of early (*MyoD* and *Myogenin*) and late (*MCK* and *MyHC*) myogenic markers in healthy myotubes with siCTL or siEMILIN1, **±**TGFβ (*N* = 3, Healthy #1). **D** RTqPCR of *EMILIN1* and of early (*MyoD* and *Myogenin*) and late (*MCK* and *MyHC*) myogenic markers in DMD myotubes with siCTL or siEMILIN1, +/- TGFβ (*N* = 3, DMD #1). **E** Immunofluorescence of MyHC (red) and nuclei staining with DAPI (blue) in healthy and DMD myotubes with siCTL or siEMILIN1. Scale bar 10 μm. A representative field is shown for each condition. **F** Quantification of myogenic and fusion index (*N* = 7 for Healthy #1, *N* = 7 for DMD #1). Data are represented as average -/+ SEM. **G** Western blot of MyoD in healthy and DMD myotubes with siCTL or siEMILIN1 in absence of TGFβ treatment. Vinculin was used as loading control (*N* = 2, Healthy #1, DMD #1, DMD #6). Vinculin was used as loading control. For all panels, data are represented with *p < 0,05, **p < 0.01; ***p < 0.001 unpaired Student’s t test.

To better characterize the role of the ECM glycoprotein EMILIN1 in skeletal muscle cells, we studied its expression levels in healthy and DMD myoblasts during myogenic differentiation into myotubes (Fig. 4A), monitoring this process with *MYOD* and *MyHC* (Myosin Heavy Chain) mRNA levels, which serve as canonical myogenic and differentiation markers (Fig. S5A). Interestingly, *EMILIN1* was more expressed in DMD proliferating myoblasts and myotubes at early stages compared to healthy controls (Fig. 4A). This finding indicates that *EMILIN1* mRNA rises at the onset of differentiation, leading to the increased EMILIN1 protein secretion observed after 3 days of differentiation (Fig. 3B).

To further investigate the role EMILIN1 during myogenic differentiation, we performed siRNA-mediated KD experiments in proliferating myoblasts (Fig. S5B) and differentiated myotubes (Fig. 4B). The efficient KD of *EMILIN1* in differentiated healthy and DMD myotubes led to a consistent and at least 2-times increase in the expression of the early master myogenic marker *MYOD*, both in basal condition and upon TGFβ treatment (Fig. 4C, D). *MYOG* expression, which acts downstream of MyoD during differentiation, was not consistently increased except when *EMILIN1* KD was performed in proliferating myoblasts (Fig. S5C, D). The expression of the intermediate myogenic marker *MCK* (muscle creatine kinase, a cytoplasmic enzyme involved in energy homeostasis) was only mildly impacted, showing almost no difference in KD DMD myotubes (Fig. 4C, D). However, the expression of the late myogenic marker *MyHC* was decreased upon *EMILIN1* KD in differentiated healthy cells (Fig. 4C) and both in healthy and DMD cells when *EMILIN1* KD was performed in proliferating myoblasts (Fig. S5C, D). The negative impact of *EMILIN1* KD on the expression of *MyHC* was confirmed by the presence of less myotubes upon *EMILIN1* depletion (Fig. 4E), as represented by the quantification of the myogenic and fusion index (Fig. 4F). To exclude that these effects were DMD mutation-dependent, we performed the same experiments on myotubes carrying a different mutation in the *DMD* gene (DMD #6). The efficient KD of *EMILIN1* led to an increase of the expression of MyoD, not only at the mRNA (Fig. S6A, B) but also at the protein level (Fig. 4G and Fig. S6C, E), without a clear impact on *MYOG*, while it reduced the expression of *MCK* and *MyHC* and results are similar with or without TGFβ (Fig. S6A, B). The inability of the cells to reach the late stages of differentiation was confirmed by a decrease in the number of multinucleated cells (Fig. S6C), as quantified by myogenic and fusion index (Fig. S6D). Interestingly, *EMILIN1* KD does not affect healthy and DMD myoblast proliferation (Fig. S7A, D).

These results reveal a role of EMILIN1 in skeletal muscle differentiation, showing that its loss leads to increased expression of early myogenic marker *MYOD* but reduced expression of the late marker *MyHC*. This reduction results in fewer multinucleated myotubes, indicating impaired late-stage differentiation. These effects are consistent across both healthy and DMD myotubes, suggesting that controlled levels of EMILIN1 are important for proper muscle cell differentiation.

### EMILIN1 regulates the expression of the fibrotic marker *SERPINE1*

**Fig. 5:**
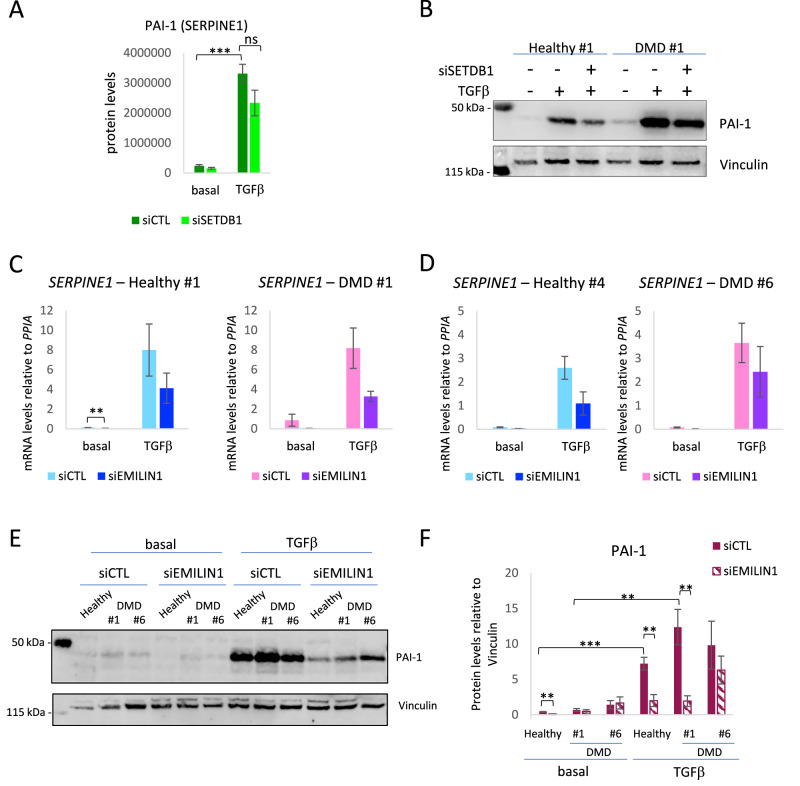
EMILIN1 KD decreases the expression of the TGFβ- and SETDB1-dependent fibrotic marker SERPINE1 in healthy and DMD myotubes. **A** MS data analysis of PAI-1 (*SERPINE1*) in DMD with siSETDB1 **±** TGFβ. Data are represented as average **±** SEM. **B** Western blot of PAI-1 in CM obtained as in Fig. 1 A from healthy and DMD myotubes **±**siSETDB1, **±**TGFβ. Vinculin was used as loading control (*N* = 4, Healthy #1, DMD #1). Quantification is shown in Fig. S8A. **C**, **D** RTqPCR of *SERPINE1* in healthy and DMD myotubes with siEMILIN1 **±**- TGFβ. as in Fig. 4B (*N* = 3, Healthy #1, DMD #1 for C and Healthy #4, DMD #6 for D). Data are represented as average -/+ SEM. **E** Western blot of PAI-1 in myotubes treated with siEMILIN1 and TGFβ as in Fig. 4B (*N* = 4, Healthy #1, DMD #1, DMD #6). Vinculin was used as loading control. **F** Quantification of the western blot 5E normalized on Vinculin (*N* = 4, Healthy #1, DMD #1 and DMD #6). Data are represented as average -/+ SEM. For all panels, data are represented with *p < 0,05, **p < 0.01; ***p < 0.001 unpaired Student’s *t* test.

To assess the role of EMILIN1 in the TGFβ-mediated fibrotic response, we focused on *SERPINE1*, a fibrotic marker we have already studied in our previous work [8]. PAI-1 (plasminogen activator inhibitor 1), the protein encoded by *SERPINE1*, was increased in the secretome of TGFβ-treated DMD myotubes, and it decreased upon SETDB1 KD (Fig. 5A). Moreover, it was enriched in the secretome of DMD myotubes compared to healthy ones, being already detected in basal condition and further increased by TGFβ (Fig. 5B, Fig. S8A). Remarkably, *SERPINE1* expression was impacted by EMILIN1. *EMILIN1* KD in differentiated healthy and DMD myotubes induced a decrease in *SERPINE1* expression both in basal condition and upon TGFβ treatment (Fig. 5C). This decreasing trend in *SERPINE1* mRNA levels was validated also upon *EMILIN1* KD in proliferating myoblasts (Fig. S8B) and in myotubes healthy #4 and DMD #6 (Fig. 5D). Notably, the levels of PAI-1, were reduced following *EMILIN1* KD (Fig. 5E, F), mirroring the changes observed at the mRNA level. This further supports the observation that reducing EMILIN1 leads to a decrease in the fibrotic marker PAI-1.

Together, these findings suggest that EMILIN1 plays a role in regulating the TGFβ-mediated fibrotic response, as indicated by the modulation of the fibrotic marker *SERPINE1*.

## Discussion

The regenerative capacity of skeletal muscle depends on the dynamic interplay between its various components, including muscle fibers and the surrounding connective tissue. This tissue operates as an integrated system, finely tuned to respond to injury and maintain muscle function [15]. While skeletal muscle is primarily known for its role in contraction, it also functions as an active endocrine organ, capable of releasing a variety of signaling factors. These factors enable muscle cells to communicate in an auto-, para-, or endocrine manner, influencing various physiological processes, including muscle regeneration [16]. Muscle regeneration is a highly coordinated process, that depends on the precise secretion of multiple factors to orchestrate tissue repair. Through the complex communication between muscle cells, immune cells and Fibro-Adipogenic Progenitors (FAPs), skeletal muscle can effectively repair itself and restore functionality. Notably, in DMD, muscle regenerating abilities are compromised. The sustained inflammation leads to TGFβ-induced fibrosis, defined as an excessive accumulation of ECM components, and to fat accumulation. Over time, functional muscle tissue is replaced with non-functional fibrotic and fatty tissue, resulting in progressive muscle loss and fibro-fatty tissue accumulation [2].

Our previous work has established that the conditioned medium from TGFβ-treated myotubes has a negative impact on myoblast differentiation and promotes fibrosis. SETDB1 KD is able to partially counteract this effect, indicating that SETDB1 potentiates the TGFβ-induced fibrosis in DMD muscles. However, even though targeting SETDB1 in postmitotic myotubes and in FAPs does not seem to have a deleterious effect on cell viability, its targeting in muscle tissue would be challenging, since the impact on other cell types, such as macrophages, or on the crosstalk between cell types, is unknown [8, 17]. Therefore, it appears crucial to identify the SETDB1/TGFβ-dependent secreted factors which could play a role in the deleterious environment of DMD.

Our data presented here describe the effect of SETDB1 regulation on the TGFβ pathway, in particular regarding the composition of the DMD secretome. The extensive mass spectrometry analysis revealed the presence of almost 3000 proteins in the secretome of DMD myotubes. The good clustering of the samples highlights that SETDB1 and TGFβ are indeed impacting the identity of the secretome. To better understand the effect of the CM described in our previous work [8], we focused on proteins that are upregulated by TGFβ, potentially contributing to impaired myoblast differentiation and enhanced fibrosis, but are downregulated upon SETDB1 KD, where the described detrimental effects are attenuated.

In addition to the changes induced by TGFβ and SETDB1, our data also provide insights into the composition of the basal DMD secretome. Notably, several secreted factors, including ECM and signaling proteins, are already present at significant levels in untreated DMD myotubes. This suggests that even in the absence of exogenous TGFβ stimulation, the DMD muscle environment is primed toward a fibrotic and regenerative-impairing profile. Interestingly, the integrated results of both secretome analyses reveal EMILIN1 among the secreted proteins. This ECM glycoprotein is not only upregulated in response to TGFβ and downregulated upon SETDB1 knockdown, but also present in the basal DMD secretome, where it shows higher levels compared to healthy controls. Moreover, EMILIN1 is overexpressed in patient biopsies, suggesting a dual mode of regulation and a sustained involvement in the pathological environment.

EMILIN1 is a protein involved in elastic and collagen fibers formation, whose role in skeletal muscle remains largely unexplored. Interestingly, EMILIN1 possesses a gC1q domain, which mediates interactions with integrins, contributing to cell adhesion and migration, and an EMI domain with seven cysteine residues, involved in TGFβ processing [10]. EMILIN1 is described as an inhibitor of the TGFβ pathway in different contexts, such as cancer and skin psoriasis [11–13]. Although *EMILIN1* knockout mice appear grossly normal, they manifest specific phenotypes including skin defects and hypertension [18]. In humans, two distinct missense mutations within the *EMILIN1* gene have been identified: c.64 G > A (p.A22T) in exon 1, associated with connective tissue disorder and sensory-motor neuropathy; and c.748 C > T (p.R250C) in exon 4, linked to distal motor neuropathy [19, 20]. However, EMILIN1 role in skeletal muscle and, specifically in DMD, is unknown.

The detection of EMILIN1 under basal conditions further supports its relevance as a constitutive component of the dystrophic secretome, whose function may be altered independently of acute TGFβ modulation. Importantly, EMILIN1 is not only detected in the basal secretome of DMD myotubes, but is also found to be upregulated in muscle tissue sections from DMD patients, reinforcing its relevance in vivo and suggesting that its role in DMD pathology extends beyond in vitro models.

**Fig. 6:**
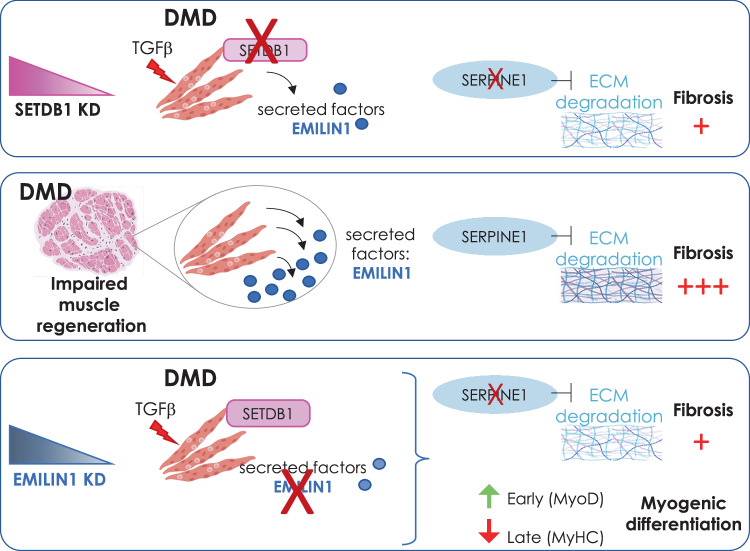
Graphical abstract. *Upper:* SETDB1 KD in DMD TGFβ-treated myotubes leads to a decrease in the expression of secreted factors associated with ECM composition and remodeling. *Middle:* The DMD impaired regenerative environment is characterized by an overexpression of EMILIN1 and of the profibrotic factor SERPINE1. *Lower:* EMILIN1 KD in DMD myotubes decreases the expression of the profibrotic marker SERPINE1 and regulates myogenic differentiation, increasing the expression of MyoD and decreasing terminal differentiation.

Here, we show that *EMILIN1* KD in myoblasts and differentiated myotubes has an impact on the differentiation process. Specifically, *EMILIN1* KD led to an increase in the early myogenic marker MyoD. This result is in accordance with experiments done in *Xenopus* where overexpression of EMILIN1 or only EMI-domain in eight cell stage embryos reduced MyoD expression and inhibited mesoderm development [11]. Despite MyoD upregulation and no impact on myoblasts proliferation, we observe a decrease in the late myogenic marker MyHC. The reduction in multinucleated myotubes upon *EMILIN1* KD further highlights the role of EMILIN1 in promoting proper muscle fiber maturation. Notably, the same pattern of differentiation impairment was observed in both healthy and DMD myotubes, suggesting that EMILIN1 regulates muscle differentiation in a similar manner regardless the presence of mutations in the *DMD* gene (Fig. 6). Whether EMILIN1 regulates myogenic differentiation through the TGFβ pathway, which is known to prevent myoblasts fusion [21], or through alternative mechanisms is yet to be explored.

Additionally, we show that *EMILIN1* KD leads to a reduction in the expression of the fibrotic and TGFβ-dependent marker *SERPINE1*. *SERPINE1* codes for the plasminogen activator inhibitor 1 (PAI-1), a protein localized in the extracellular space which plays critical roles in response to skeletal muscle injury and in myopathies. PAI-1 contributes to the inhibition of ECM degradation, and excessive PAI-1 level promotes fibrosis and results in impaired skeletal muscle regeneration [22].

Given the inhibitory role of EMILIN1 on TGFβ signaling in other contexts, it may seem counterintuitive that its reduction would lead to decreased fibrosis, potentially improving the DMD environment. However, this finding might be context-dependent and further studies are needed to elucidate the interplay between EMILIN1 and the TGFβ pathway in DMD. One possible explanation is that EMILIN1-mediated inhibition of TGFβ signaling may be less relevant in the context of DMD. Indeed, we observed that *EMILIN1* knockdown did not alter *TGF*β mRNA (data not shown). Alternatively, EMILIN1 may regulate *SERPINE1* indirectly, either by suppressing a negative regulator or by enhancing an upstream activator of *SERPINE1* expression or activity. Moreover, it would be interesting to study the role of EMILIN1 in FAPs, key mediators of the fibrotic response. Such studies could provide valuable insights into how this secreted factor contributes to the DMD phenotype.

In summary, these findings reveal critical insights into the interplay between SETDB1, TGFβ signaling, and the fibrotic response in DMD. The data highlight the role of SETDB1 in modulating the secretome of DMD myotubes and provide a rich list of factors that could contribute to the pathological environment characteristic of DMD. Importantly, our data show that several components of this secretome, including EMILIN1, are already present in the basal dystrophic state and are upregulated in patient muscle sections, suggesting a pre-existing and persistent alteration of the secretory profile in DMD muscle. Among these factors, EMILIN1 emerged as an important player involved in the regulation of myogenic differentiation and fibrosis (Fig. 6). While EMILIN1 targeting appears promising for reducing fibrosis, it does not enhance terminal myotube differentiation. Future studies using three-dimensional, disease-relevant models [23] will be essential to determine whether the antifibrotic effects of EMILIN1 modulation can ultimately translate into functional improvement, despite potential impacts on muscle differentiation. Alternatively, combinatorial therapeutic strategies that couple EMILIN1-based antifibrotic interventions with complementary approaches to enhance myogenic differentiation may provide a more effective means of restoring muscle integrity. Collectively, our results suggest that SETDB1-dependent secreted factors can influence both myoblast differentiation and fibrotic signaling, offering novel potential targets for therapeutic intervention in DMD.

## Materials and methods

### Human muscle samples

Human muscle samples from healthy controls and Duchenne muscular dystrophy patients (Table 1) were obtained via the Myobank-AFM, affiliated with EurobioBank, with the subject’s agreement based on an informed consent form, in accordance with European recommendations and French legislation (authorization AC-2019-3502).

**Table 1:** Muscle biopsies.

Status	Muscle	Dystrophin mutation	Age	Name
Healthy	Paravertebral		19	Healthy #B1
			18	Healthy #B2
			17	Healthy #B3
	Dorsal		17	Healthy #B4
			15	Healthy #B5
	Quadriceps		10	Healthy #B6
			19	Healthy #B7
DMD	Paravertebral	mutation intron 66: c.IV566+1 G > A	15	DMD #B1
		Stop mutation c.10141 C > T, pArg3381X	15	DMD #B2
		del 8-43	12	DMD #B3
		c.2281_2285delGAAA;c.Glu761SerfsX10	12	DMD #B4
		del 3-9	12	DMD #B5
		del 10-41	16	DMD #B6
		c.6746dupA	12	DMD #B7
	Fascia lata	ND	8	DMD #B8

*ND* not determined.

### Cell culture

Human control and DMD immortalized myoblasts were obtained from the MyoLine platform for immortalization of human cells from the Institute of Myology (Paris, France) (Table 2). Immortalization was performed as described in [14], using human Telomerase-expressing (hTERT) and Cyclin-Dependent Kinase 4 (CDK4)–expressing vectors. They were cultured on gelatin-coated plates for all the experiments except for the preparation of the secretome for the healthy *versus* DMD comparison. Immortalized myoblasts were cultured in growth medium (GM) composed of Dulbecco’s modified Eagle’s medium (DMEM) high glucose GlutaMAX (Invitrogen, 61965-026)/Medium 199 (Invitrogen, 41150020) 4:1 mixture, supplemented with 20% fetal bovine serum (Dutscher, 500105M1M batch S00G0), Fetuin (25 μg/ml; Sigma-Aldrich, F3385), Insulin (5 μg/ml; Sigma-Aldrich, I9278), basic Fibroblast Growth Factor (0.5 ng/ml; Sigma-Aldrich, SRP3043), human Epidermal Growth Factor (5 ng/ml; Sigma-Aldrich, SRP3027), and dexamethasone (0.2 μg/ml; Sigma-Aldrich, D4902).

**Table 2:** Cell lines.

Status	Muscle	Dystrophin mutation	Age	Name	Name in PRIDE
Healthy	Paravertebral		16	Healthy #1	CTL-12
			13	Healthy #2	CTL-13
			12	Healthy #3	CTL-14
	Fascia lata tensor		20	Healthy #4	CTL-1
	Gracilis		25	Healthy #5	CTL-2
DMD	Paravertebral	Del 45-52	13	DMD #1	
		Del 51-54	15	DMD #2	D3-4
		Mutation intron 70: C.IVS70 + 1 G > A	17	DMD #3	D3-3
	Spinal	Del 45	14	DMD #4	D3-1
	Dorsal	Duplication exon 10 to 11	14	DMD #5	D3-2
	Quadriceps	stop mutation exon 59: c.8713 C > T, p.Arg2905X	11	DMD #6	

### Myogenic differentiation of human immortalized myoblasts

Human control and DMD-immortalized myoblasts were grown at >80% confluence, and the medium was switched for DM media. The DM media was composed of DMEM with gentamycin for the basal DMD vs control secretome comparison and of DMEM with insulin (10μg/mL; Sigma-Aldrich, I9278) for all the other experiments, as described in [8].

### Myoblast and myotube transfection with siRNAs

Proliferating myoblasts (cultured in GM) or two-day-differentiated myotubes (cultured in DM) were transfected with siRNAs at a final concentration of 70 nM, using the Lipofectamin RNAiMAX transfection agent (Invitrogen, 13778100). Cells were kept for 2 days in a transfection medium before switching to fresh media. We used the ON-TARGETplus Human SETDB1 siRNA (Dharmacon, L-020070-00-0010); EMILIN1 siRNA (Sigma-Aldrich, SASI_Hs01_00218611), and the scrambled control siRNA (Sigma-Aldrich, F: CAUGUCAUGUGUCACAUCUC[dT][dT], R: GAGAUGUGACACAUGACAUG[dT][dT]).

### Immunofluorescence

Human myoblasts were grown on gelatin-coated coverslips. They were fixed with 4% paraformaldehyde for 20 min, and they were saturated with 50 mM NH_4_Cl for 10 min at room temperature (RT). Then, they were permeabilized with 0.5% Triton X-100 for 5 min. Cells were washed three times with PBS between each step. Cells were next incubated with primary antibodies at the concentration indicated in the antibody list below. Alexa Fluor 555 was used as secondary antibodies (Invitrogen, 115-165-207). Nuclei were counterstained with 4′,6-diamidino-2-phenylindole (DAPI). Cells were washed three times with PBS-Tween 0.1% FBS 2% after each antibody incubation.

Images were acquired with a Leica DMI-6000B microscope and analysis was performed using Fiji software

For immunostaining of muscle biopsies, cryosections were fixed in 4% paraformaldehyde for 10 min at RT and incubated with PBS containing 2% FBS and 0,5% triton for 30 min at RT. Incubation with primary antibodies was performed at RT for 1 h and cryosections were incubated with appropriate fluorescent secondary antibodies (Life technologies) at 1/400 at RT for 45 min. Nuclei were stained with Hoechst. Widefield fluorescence images were taken with an Axio Observer 7 microscope (Zeiss) equipped with a motorized stage coupled to an Orca Flash 4 Camera (Hamamatsu) and driven by the Zen software (Zeiss). The antibodies used are listed in Table 3.

**Table 3:** Antibodies.

Antibody	Reference	Use and dilution
Myosin heavy chain, MYH1E (mouse IgG2b)	DSHB MF20	Immunofluorescence (1:10)
PAI1 (rabbit)	Proteintech, 13801-1-AP	Western blot (1:1000)
MyoD (rabbit)	Cell Signaling Technology, 13812	Western blot (1:1000)
Vinculin (mouse IgG1)	Sigma-Aldrich, V9131	Western blot (1:500)
Emilin1 (rabbit)	Kind gift from Paola Spessotto, As-556	Immunofluorescence (1:1000)
Emilin1 (rabbit)	Abcam, AB185953	Immunofluorescence (1:100)
Laminin (rabbit)	Dako, Z0097	Immunofluorescence (1:400)

### RNA extraction and reverse transcription quantitative PCR (RTqPCR)

Total RNA from cells was extracted using RNeasy Micro Kit (Qiagen, 74004) following the manufacturer’s procedures. Deoxyribonuclease (Qiagen, 79254) treatment was performed to remove residual DNA. One microgram of total RNA was reverse-transcribed with a High-Capacity cDNA Reverse Transcription Kit (Applied Biosystems, 4368813). Real-time quantitative PCR was performed to analyze relative gene expression levels using SYBR Green GoTaq® qPCR Master Mix (Promega, A6002) following the manufacturer’s indications. Relative expression values were normalized to the housekeeping gene mRNA *PPIA*.

For human frozen muscle sections, RNA was extracted using Trizol reagent (Invitrogen) according to the manufacturer’s instructions. RNA was reverse transcribed using M-MLV (Invitrogen) according to the manufacturer’s instructions. Quantitative polymerase chain reaction (qPCR) was carried out using SYBR Green Mastermix (Roche Applied Science) in a Applied Biosystems QuantStudio 6 Pro (Thermofisher scientific) with the following cycling protocol: 8 min at 95 °C; followed by 50 cycles at 95°C for 15 s (s), 60 °C for 15 s and 72 °C for 15 s, and a final step consisting of 5 s at 95 °C and 1 min at 65 °C. The specificity of the PCR product was evaluated by melting curve analysis using the following program: 65 °C to 97 °C with a 0.11 °C/s increase and gene expression levels were normalized to human RPLP0 and quantified with the 2–ΔΔCt method. Primers are listed in Table 4.

**Table 4:** Primers.

Targets	Forward	Reverse
*MYOGENIN*	AATGCAGCTCTCACAGCGCCTC	TCAGCCGTGAGCAGATGATCC
*MyHC*	CTGTTGCAGTTTCTCATTGGTG	CCAGGCAGTACTTCATTGGG
*CTGF*	TGTGCACCGCCAAAGAT	GCACGTGCACTGGTACTT
*SERPINE1*	TCCACAAATCAGACGGCAGC	TCGTAGTAATGGCCATCGGG
*THBS1*	CTGGCCCAATGAGAACCTGG	GCCCTGAGTTGGGAAGGTTG
*MYOD*	TGCTCCGACGGCATGATGGACTA	TTGTAGTAGGCGCCTTCGTAGCAGTT
*MCK*	TGGAGAAGCTCTCTGTGGAAGCTC	TCCGTCATGCTCTTCAGAGGGTAGTA
*EMILIN1*	GCGATGACTGTGCTGAGAGT	TGACTCCCCAGGACCTTCT
*IL11*	CTCCTGATGTCCCGCCTG	CAGTCAAGTGTCAGGTGCAG
*MMP14*	CGTTGTCTCCTGCTCCCC	CTGTGTGTGGGTACGTAGGT
*PPIA*	GGTGACTTCACACGCCATA	ACAAGATGCCAGGACCCGT
*RPLP0*	TGGTCATCCAGCAGGTGTTCGA	ACAGACACTGGCAACATTGCGG

### Western blot

Cells were lysed in RIPA buffer [20 mM tris (pH 7.65), 150 mM NaCl, 0.1% SDS, 0.25% NaDoc, and 1% NP-40] supplemented with protease inhibitor 1× (Sigma-Aldrich, SIGMAFAST S8830) and phosphatase inhibitor 1× (Sigma-Aldrich, P5726-5ML). Cell lysates were sonicated at 4 °C for 10 min (30 s ON, 30 s OFF) at medium frequency (Bioruptor Diagenode). Then, the lysates were centrifugated for 10 min at 4 °C at 10,000 rcf and the supernatants were kept as the samples. Extracts were resolved on pre-cast polyacrylamide gel cassettes (NuPAGE 4 to 12% Bis-Tris) (Invitrogen NP0335BOX, NP0336BOX) and 1× NuPAGE MOPS SDS Running Buffer (Invitrogen, NP0001) and transferred into nitrocellulose membrane (Sigma-Aldrich, Amersham, 10600002) in 20 mM phosphate transfer buffer (pH 6.7). The membrane was blocked in 5% skim milk in PBST buffer (1× PBS, 0.2% Tween 20) and incubated overnight at 4 °C with the primary antibody (Table 3). Membranes were washed twice for 5 min in PBST, incubated with appropriate secondary antibody IRDye (Li-Cor, IRDye 800CW, IRDye 680RD) in PBST, washed twice for 10 min in PBST, once for 10 min in PBS, and then imaged on Odyssey Imaging System. Uncropped western blot images are provided as supplemental material (see Original Data file)

### Collection and preparation of conditioned media for WB and analysis by mass spectrometry (MS)

Human myoblasts were cultured and differentiated as described above (serum-free DM). On day 1 of differentiation, cells were transfected with siSETDB1. Two days post transfection, cells were treated with TGFβ (20 ng/mL; Peprotech, 100-21C-10UG) for 5 hours. Then, cells were washed five times with PBS before putting fresh DM. The CM was collected 72 h later and filtered using a 0.2 μm filter to ensure removal of any dead cells. The CM was concentrated using spin columns with 10 kDa cut off (Millipore Amicon Ultra, UFC5010) performing a centrifugation of 10 min at 10000 RCF, 4 °C. The protein content of the CM was measured with BCA assay (ThermoFisher, Pierce BCA Protein Assay Kit, 23227). The concentrated CM was analyzed by WB (as described above) or by MS (10 μg of proteins per condition were analyzed as follows).

### Mass spectrometry

Mass spectrometry LC-MS/MS analyses were performed on two sites: at Proteom’IC, the proteomics core facility of Institut Cochin in Paris (https://institutcochin.fr/plateformes/proteomic) for the basal DMD vs control comparison and at ProteoSeine, the proteomics facility of Institut Jacques Monod in Paris (https://www.ijm.fr/plateformes-et-plateaux-techniques/proteoseine/) for the TGFβ and SETDB1 study. These MS proteomics data have been deposited to the ProteomeXchange Consortium via the PRIDE partner repository with the dataset identifier PXD068544 and PXD074360.

#### Samples preparation prior to LC-MS/MS analysis

Protein samples from both protocols underwent denaturation, reduction, alkylation, enzymatic digestion and peptide purification prior to LC-MS/MS analysis. However, specific conditions were adapted to the biological context and experimental constraints.

For the DMD secretome, proteins were extracted in a lysis buffer containing 400 mM triethylammonium bicarbonate, 4% SDS, 100 mM chloroacetamide, and 20 mM tris(2-carboxyethyl)phosphine, followed by heating at 95 °C for 5 min. Protein digestion was performed using trypsin (Promega) in S-Trap Micro Spin Columns (Protifi), following the manufacturer’s instructions. The resulting peptides were dried by SpeedVac and resuspended in 10 µL of 0.1% trifluoroacetic acid (TFA) with 10% acetonitrile (ACN). In cases of column clogging, additional clean-up was performed using strong cation exchange (SCX) membranes (3 M Empore) [24]. Peptides were resuspended in 2% TFA, passed through SCX disks packed in modified P200 tips, washed with TFA-based buffers, and eluted with 5% ammonium hydroxide in 80% ACN. The eluates were dried and reconstituted in the same loading buffer as above.

For the SETDB1-TGFβ secretome, a six-time volume of cold acetone (−20 °C) was added to a sample volume containing 10 µg of protein extracts. Vortexed tubes were incubated overnight at −20 °C then centrifuged for 10 min at 11,000 rpm, 4 °C. The protein pellets were dissolved in urea 8 M - NH_4_HCO_3_ 25 mM buffer. Samples were then reduced with DTT 10 mM and alkylated with IAA 20 mM. After a 16-fold dilution in NH_4_HCO_3_, samples were then digested overnight at 37 °C by a mixture of trypsin/Lys C (1/20 Enzyme/Substrate ratio). The digested peptides were loaded and desalted on Evotips provided by Evosep one (Odense, Denmark) according to manufacturer’s procedure.

#### LC-MS/MS acquisition

Peptides from both protocols were analyzed using a timsTOF Pro 2 mass spectrometer (Bruker Daltonics, Bremen, Germany), operated in data-dependent acquisition with Parallel Accumulation-Serial Fragmentation (PASEF) mode [25], and coupled either to a Dionex U3000 RSLC nano-LC system (DMD samples) or to an Evosep one system (Evosep, Odense, Denmark) operating with the 30SPD method developed by the manufacturer (SETDB1- TGFβ samples). Chromatographic separation was performed on C18 reverse-phase columns under nanoflow conditions, using a binary solvent system consisting of water with 0.1% formic acid (solvent A) and acetonitrile with 0.1% formic acid (solvent B). For the SETDB1-TGFβ samples, peptides were separated using a 0.15 × 150 mm C18 analytical column (1.9 µm particles, EV-1106; Evosep) operated at 40 °C and 500 nL/min under the manufacturer’s 30 samples-per-day method. A 44-minute linear gradient was applied, resulting in a total run time of 48 min. In contrast, DMD and healthy samples were analyzed using an Aurora C18 column (75 µm × 25 cm, 1.7 µm particles, IonOpticks) over a 2-h gradient, ranging from 98% solvent A to 35% solvent B. For SCX-cleaned samples, a shorter multistep gradient was used (10% B in 14 min, 20% B in 25 min, 40% B in 38 min). In all cases, the timsTOF instrument operated in positive ion mode, with precursor scans acquired in the 100–1700 m/z range. Ion mobility spectrometry (TIMS) was enabled, with accumulation and ramp times set between 166 ms and 180 ms. The ion mobility range was set to 1/K₀ = 0.6–1.6 Vs/cm² (DMD) or 0.75–1.25 Vs/cm² (SETDB1-TGFβ). PASEF cycles included 6 (SETDB1-TGFβ) or up to 10–12 MS/MS scans (DMD), achieving near-complete duty cycles.

Precursor ions were selected based on intensity thresholds (>1000 a.u.) and dynamically excluded for 0.4 s (DMD) or 0.8 min (SETDB1-TGFβ). They were re-sequenced until reaching a target intensity of 20,000 a.u. for DMD or 16,000 a.u. for SETDB1-TGFβ. Quadrupole isolation was synchronized with TIMS elution profiles, and collision energy was ramped based on ion mobility in all cases.

#### MS data analysis

Data from the DMD secretome were processed using MaxQuant (version 1.6.17.0)) [26]. Spectra were searched against a database combining SwissProt human sequences (release 2020-03) with known contaminants from MaxQuant and the cRAP repository. Trypsin was set as the proteolytic enzyme, allowing for up to two missed cleavages. Carbamidomethylation of cysteines was defined as a fixed modification, while oxidation of methionine and N-terminal acetylation were considered variable. The false discovery rate (FDR) was controlled below 1% at both peptide and protein levels. The “match between runs” feature was enabled to improve peptide identification across samples.

For the SETDB1-TGFβ secretome, MS raw files were processed using PEAKS Online X (build 1.8, Bioinformatics Solutions Inc.). Data were searched against the SwissProt database (https://www.expasy.org/resources/uniprotkb-swiss-prot). Parent mass tolerance was set to 20 ppm, with fragment mass tolerance at 0.05 Da. Specific tryptic cleavage was selected and a maximum of 2 missed cleavages was authorized. For identification, the following post-translational modifications were included: Acetylation (Protein N-term), oxidation, deamidation as variables and carbamidomethylation as fixed. Identifications were filtered based on a 1% false discovery rate threshold at both peptide and protein group levels. Label free quantification was performed using the PEAKS Online X quantification module, allowing a mass tolerance of 10 ppm, a CCS error tolerance of 0.02 and a 0.5-min retention time shift tolerance for match between runs. Protein abundance was inferred using the top N peptide method and TIC was used for normalization. Multivariate statistics on proteins were performed using Qlucore Omics Explorer 3.8 (Qlucore AB, Lund, *SWEDEN*). A positive threshold value of 1 was specified to enable a log2 transformation of abundance data for normalization i.e., all abundance data values below the threshold will be replaced by 1 before transformation. The transformed data were finally used for statistical analysis i.e., evaluation of differentially present proteins between two groups using a Student’s bilateral t-test. A p-value better than 0.01 was used to filter differential candidates.

Functional enrichment analysis was performed using the functional annotation tool DAVID (Database for Annotation, Visualization, and Integrated Discovery, https://davidbioinformatics.nih.gov/home.jsp) [27], and p-values were adjusted for multiple testing using the Benjamini–Hochberg false discovery rate.

### Statistical analyses

Statistical analyses were carried out using Excel or GraphPad Prism (version 8.0, GraphPad Software Inc.). Data are represented as means ± SEM or mean ± SD as described in the figure legend. Two-tailed *t* test was used for statistical analysis; *P < 0.05, **P < 0.01, and ***P < 0.001.

## Supplementary information

supp material

Figure S1

Figure S2

Figure S3

Figure S4

Figure S5

Figure S6

Figure S7

Figure S8

Tables S1-S5

Uncropped western blots

## Data Availability

All data needed to evaluate the conclusions in the paper are present in the paper and/or the Supplementary Materials. Data corresponding to the experiments described in this study are deposited on ProteomeXchange Consortium via the PRIDE partner repository (PRIDE identifiers PXD068544 and PXD074360).
