# Supplementary material for: EMILIN1 emerges as a TGFβ/SETDB1-regulated secreted biomarker in Duchenne muscular dystrophy
Source: Cell Death Dis. 2026 May 9;17(1):611. doi: 10.1038/s41419-026-08825-8 (PMC13323359; doi:10.1038/s41419-026-08825-8)
Supplement: Supplementary file 1 — supp material [file 41419_2026_8825_MOESM1_ESM.pdf]

SUPPLEMENTARY MATERIALS: 8 Supplementary figures, 5 supplementary tables and 1 original data file of uncropped western blots (3 pages).

Figure S1. Detailed proteomic analysis of the TGFβ- and SETDB1-regulated secretome of DMD myotubes.

A. Complete heatmap (ANOVA, p-value < 0.001) from the secretome analysis described in Fig.1.

B. DAVID bioinformatics functional annotation chart (Sherman et al., 2022) using uniprotKB keywords (biological process, cellular component, molecular function and ligand) using up and unique proteins from siCTL+TGFβ versus siCTL comparison.

C. DAVID bioinformatics functional annotation chart (Sherman et al., 2022) using uniprotKB keywords (biological process, cellular component, molecular function and ligand) using up and unique protein from siSETDB1+TGFβ versus siSETDB1 comparison.

For B and C : Color intensity reflects enrichment significance after multiple testing correction using the Benjamini–Hochberg false discovery rate as implemented in DAVID Bioinformatics.

Figure S2. Analysis of SETDB1-dependent targets emerged from the proteomic analysis of the secretome of DMD myotubes.

A. MS data analysis of MMP14 (matrix metalloproteinase-14), ADAMTS4 (a disintegrin and metalloproteinase with thrombospondin motif 4), COL1A2 (collagen type I alpha 2), IL11 (interleukin 11). Data are represented as average +/-SEM.

B. RTqPCR of *MMP14* and *IL11* (N=3, DMD #1). Data are represented as average +/-SEM.

For all panels, data are represented with *p<0,05, **p<0.01; ***p<0.001 unpaired Student's t test.

Figure S3. Detailed proteomic analysis of the DMD myotubes.

Detailed heatmap from the secretome analysis described in **Fig. 2D**.

Figure S4. Common targets of the basal DMD and TGFβ–SETDB1 secretome

A. MS data analysis of ARCN (Coatomer subunit delta), NEXN (Nexilin), ADAM10 (Disintegrin and metalloproteinase domain-containing protein 10), SSC5D (Soluble scavenger receptor cysteine-rich domain-containing protein), NES (Nestin) in SETDB1 KD +/- TGFβ. Data are represented as average +/-SEM.

B. MS data analysis of ARCN (Coatomer subunit delta), NEXN (Nexilin), ADAM10 (Disintegrin and metalloproteinase domain-containing protein 10), SSC5D (Soluble scavenger receptor cysteine-rich domain-containing protein), NES (Nestin) in healthy and DMD secretome. Data are represented as average +/-SEM.

C. Immunostaining of EMILIN1 (Ab As556) (red), Laminin (gray), and Dapi (blue) on human muscle biopsies of healthy and DMD patients, scale bar = 50µm.

For all panels, data are represented with *p<0,05, **p<0.01; ***p<0.001 unpaired Student's t test.

Figure S5. EMILIN1 KD regulates myogenic differentiation by promoting early markers and inhibiting late differentiation in healthy and DMD myoblasts and myotubes.

A. RTqPCR of *MyoD* and *MyHC* in healthy and DMD proliferating myoblasts and differentiated myotubes (N=4, Healthy #1 and DMD #1). Data are represented as average +/-SEM.

B. Diagram of experimental design. Proliferating myoblasts were transfected with siRNAs scrambled (siCTL) or against EMILIN1 (siEMILIN1) for 2 days. Next, they were put in differentiation medium for 2 days and then treated or not with TGFβ at 20ng/mL for 24h.

C. RTqPCR of *EMILIN1* and of early (*MyoD* and *Myogenin*) and late (MCK and MyHC) myogenic markers in healthy myotubes with siCTL or siEMILIN1, +/- TGFβ (N=3, Healthy #1). Data are represented as average -/+ SEM.

D. RTqPCR of *EMILIN1* and of early (*MyoD* and *Myogenin*) and late (*MCK* and *MyHC*) myogenic markers in DMD myotubes with siCTL or siEMILIN1, +/- TGFβ (N=3, DMD #1). Data are represented as average -/+ SEM.

For all panels, data are represented with *p<0,05, **p<0.01; ***p<0.001 unpaired Student's t test.

Figure S6. EMILIN1 KD regulates myogenic differentiation by promoting early markers and inhibiting late differentiation in healthy and DMD myoblasts and myotubes, independently from the mutation in the *DMD* gene.

A. RTqPCR of *EMILIN1* and of early (*MyoD* and *Myogenin*) and late (*MCK* and *MyHC*) myogenic markers in healthy #4 myotubes, collected accordingly to the experimental design Figure 3B (N=3). Data are represented as average +/-SEM.

B. RTqPCR of *EMILIN1* and of early (*MyoD* and *Myogenin*) and late (*MCK* and *MyHC*) myogenic markers in DMD #6 myotubes, collected accordingly to the experimental design Figure 3B (N=3). Data are represented as average +/-SEM.

C. Representative immunofluorescence of Healthy #1 and DMD #6 myotubes with siCTL or siEMILIN1 of MyHC (red), MyoD (green) and nuclei staining with DAPI (blue). Scale bar 10 μm. A representative field is shown for each condition.

D. Quantification of myogenic and fusion index (N=7, Healthy #1 and DMD #6).

E. Quantification of MyoD signal measured by IF in Healthy #1 and DMD #6 myotubes with siCTL or siEMILIN1 (N=5). Data are represented as average -/+ SEM.

For all panels, data are represented with *p<0,05, **p<0.01; ***p<0.001 unpaired Student's t test.

Figure S7. EMILIN1 knockdown does not impact myoblasts proliferation.

A. Diagram of experimental design. Myoblasts were transfected 24h after plating and counted 72h after transfection.

B. RTqPCR of *EMILIN1* showing the KD efficiency in Healthy #1, DMD #1 and DMD #6 (N=1).

C. Light microscopy images of Healthy #1, DMD #1 and DMD #6 with siCTL or siEMILIN1.

D. Quantification of the number of cells upon EMILIN1 KD (N=5 to 7), Healthy #1, DMD #1 and DMD #6). Scale bar 10μm. Data are represented as average -/+ SEM.

For all panels, data are represented with *p<0,05, **p<0.01; ***p<0.001 unpaired Student's t test.

Figure S8. EMILIN1 KD reduces the expression of the TGFβ- and SETDB1- dependent fibrotic marker SERPINE1 in healthy and DMD myoblasts.

A. Quantification of the western blot of Fig. 5B normalized on Vinculin (N=4, Healthy#1 and DMD #1). Data are represented as average -/+ SEM.

B. RTqPCR of *SERPINE1* in healthy and DMD myoblasts +/- siEMILIN1, +/- TGFβ as in figure S4B (N=3, Healthy #1 and DMD #1). Data are represented as average +/-SEM.

For all panels, data are represented with *p<0,05, **p<0.01; ***p<0.001 unpaired Student's t test.

Table S1: ANOVA analysis of all proteins identified from DMD secretome with SETDB1 KD and TGFβ treatment.

Table S2: T-test analysis with siSETDB1 vs siSETDB1 +TGFβ (p-value <0.01).

Table S3: T-test analysis with siCTL vs siSETDB1 (p-value <0.01).

Table S4: T-test analysis with siCTL vs siCTL+TGFβ (p-value <0.01).

Table S5: T-test analysis with siCTL+TGFβ vs siSETDB1+TGFβ (p-value <0.01).

Original Data: Uncropped western blots (3 pages).
