# Supplementary material for: EMILIN1 emerges as a TGFβ/SETDB1-regulated secreted biomarker in Duchenne muscular dystrophy
Source: Cell Death Dis. 2026 May 9;17(1):611. doi: 10.1038/s41419-026-08825-8 (PMC13323359; doi:10.1038/s41419-026-08825-8)
Supplement: Supplementary file 2 — Figure S1 [file 41419_2026_8825_MOESM2_ESM.pdf]

Figure S1

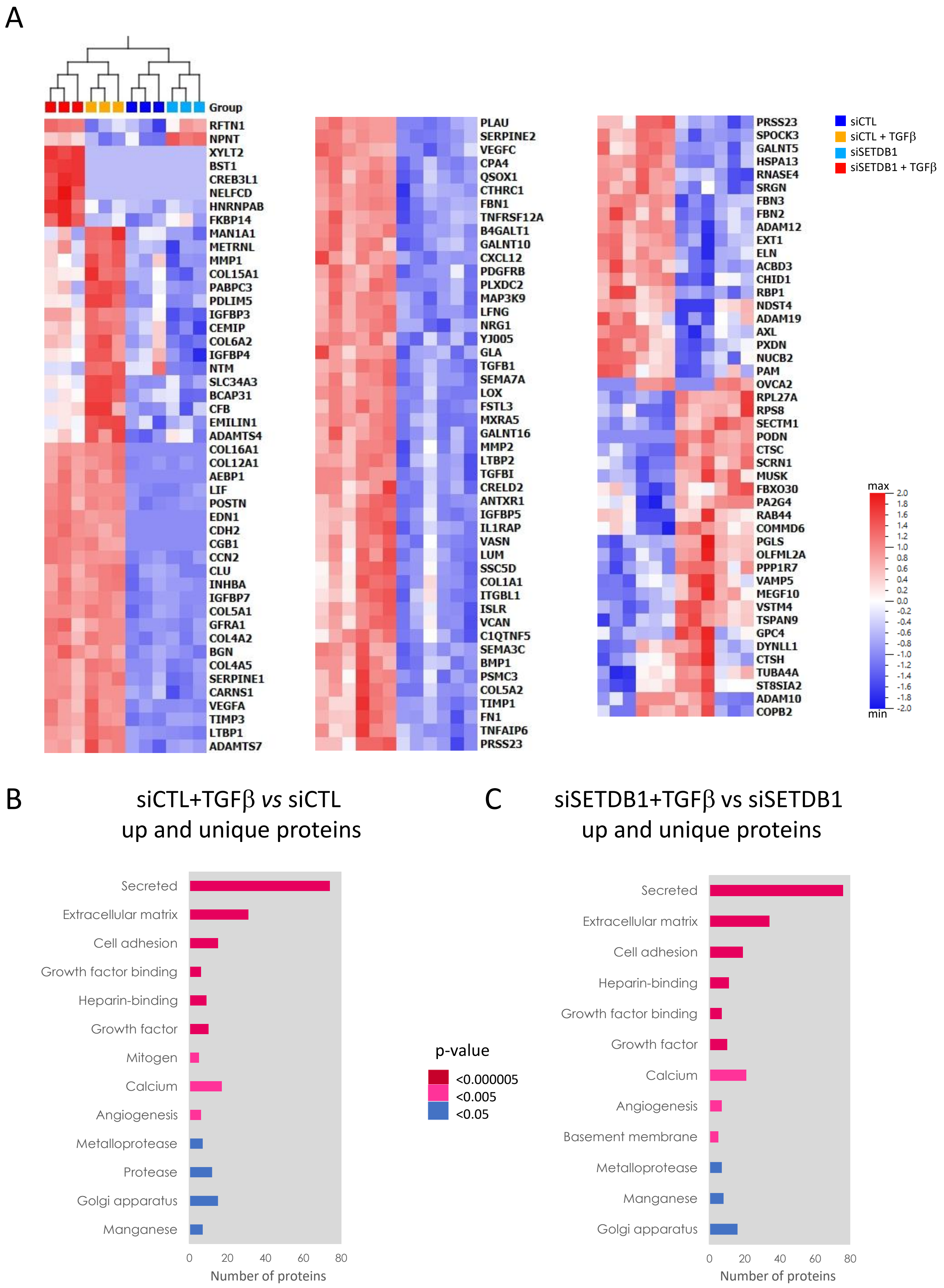

A
Group
RFTN1
NPNT
XYLT2
BST1
CREB3L1
NELFCD
HNRNPAB
FKBP14
MAN1A1
METRNL
MMP1
COL15A1
PABPC3
PDLIM5
IGFBP3
CEMIP
COL6A2
IGFBP4
NTM
SLC34A3
BCAP31
CFB
EMILIN1
ADAMTS4
COL16A1
COL12A1
AEBP1
LIF
POSTN
EDN1
CDH2
CGB1
CCN2
CLU
INHBA
IGFBP7
COL5A1
GFRA1
COL4A2
BGN
COL4A5
SERPINE1
CARNS1
VEGFA
TIMP3
LTBP1
ADAMTS7
PLAU
SERPINE2
VEGFC
CPA4
QSOX1
CTHRC1
FBN1
TNFRSF12A
B4GALT1
GALNT10
CXCL12
PDGFRB
PLXDC2
MAP3K9
LFNG
NRG1
YJ005
GLA
TGFB1
SEMA7A
LOX
FSTL3
MXRA5
GALNT16
MMP2
LTBP2
TGFBI
CRELD2
ANTXR1
IGFBP5
IL1RAP
VASN
LUM
SSC5D
COL1A1
ITGBL1
ISLR
VCAN
C1QTNF5
SEMA3C
BMP1
PSMC3
COL5A2
TIMP1
FN1
TNFAIP6
PRSS23
PRSS23
SPOCK3
GALNT5
HSPA13
RNASE4
SRGN
FBN3
FBN2
ADAM12
EXT1
ELN
ACBD3
CHID1
RBP1
NDST4
ADAM19
AXL
PXDN
NUCB2
PAM
OVCA2
RPL27A
RPS8
SECTM1
PODN
CTSC
SCRN1
MUSK
FBXO30
PA2G4
RAB44
COMMD6
PGLS
OLFML2A
PPP1R7
VAMP5
MEGF10
VSTM4
TSPAN9
GPC4
DYNLL1
CTSH
TUBA4A
ST8SIA2
ADAM10
COPB2
siCTL
siCTL + TGFβ
siSETDB1
siSETDB1 + TGFβ
max
2.0
1.8
1.6
1.4
1.2
1.0
0.8
0.6
0.4
0.2
0.0
-0.2
-0.4
-0.6
-0.8
-1.0
-1.2
-1.4
-1.6
-1.8
-2.0
min
B
siCTL+TGFβ *vs* siCTL
up and unique proteins
Secreted
Extracellular matrix
Cell adhesion
Growth factor binding
Heparin-binding
Growth factor
Mitogen
Calcium
Angiogenesis
Metalloprotease
Protease
Golgi apparatus
Manganese
0
20
40
60
80
Number of proteins
p-value
<0.000005
<0.005
<0.05
C
siSETDB1+TGFβ vs siSETDB1
up and unique proteins
Secreted
Extracellular matrix
Cell adhesion
Heparin-binding
Growth factor binding
Growth factor
Calcium
Angiogenesis
Basement membrane
Metalloprotease
Manganese
Golgi apparatus
0
20
40
60
80
Number of proteins
