# Supplementary figures and images for: EMILIN1 emerges as a TGFβ/SETDB1-regulated secreted biomarker in Duchenne muscular dystrophy
Source: Cell Death Dis. 2026 May 9;17(1):611. doi: 10.1038/s41419-026-08825-8 (PMC13323359; doi:10.1038/s41419-026-08825-8)

Figure S2

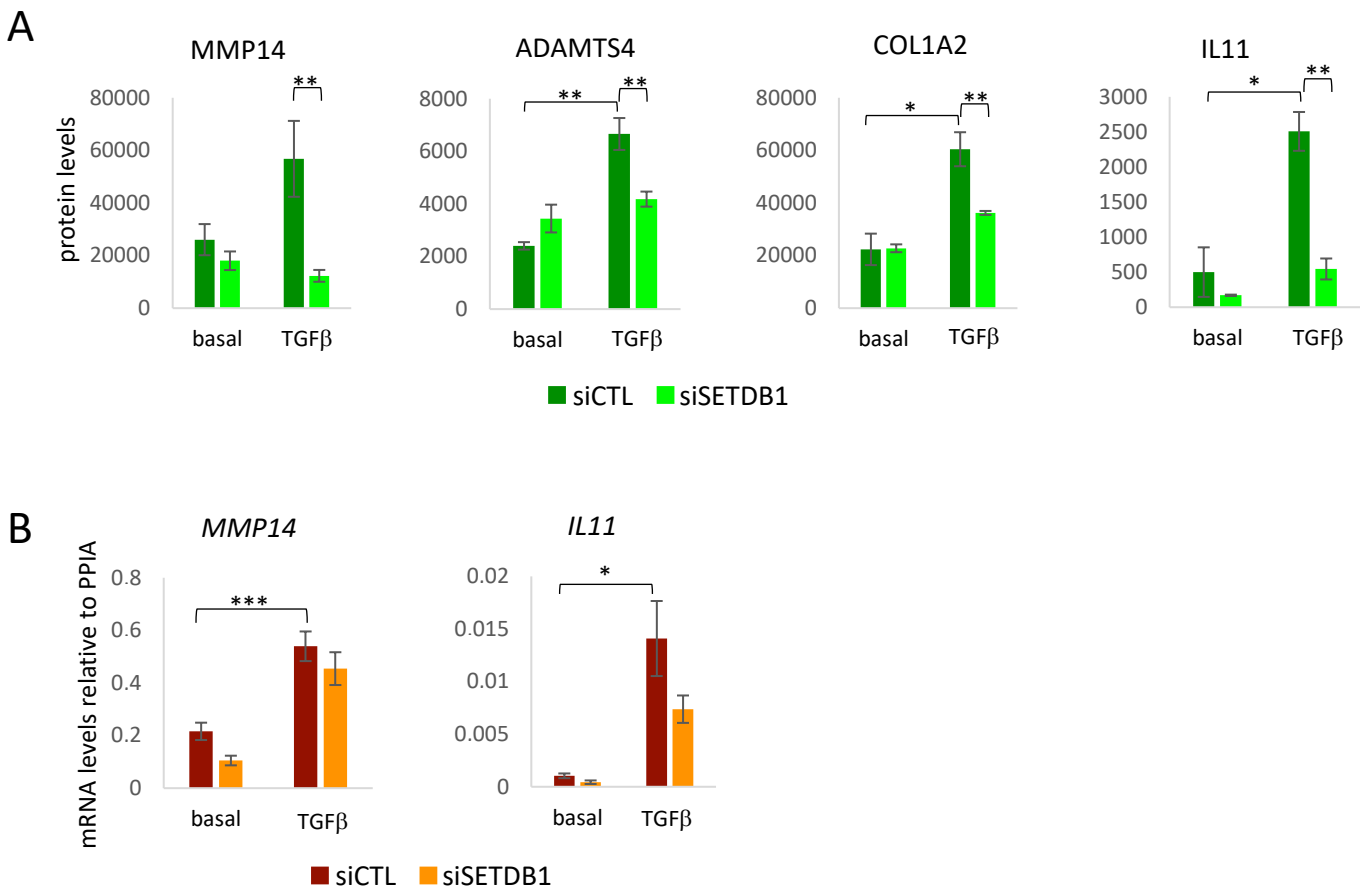

Supplement: Supplementary file 3 — Figure S2 [file 41419_2026_8825_MOESM3_ESM.pdf]

Figure S3

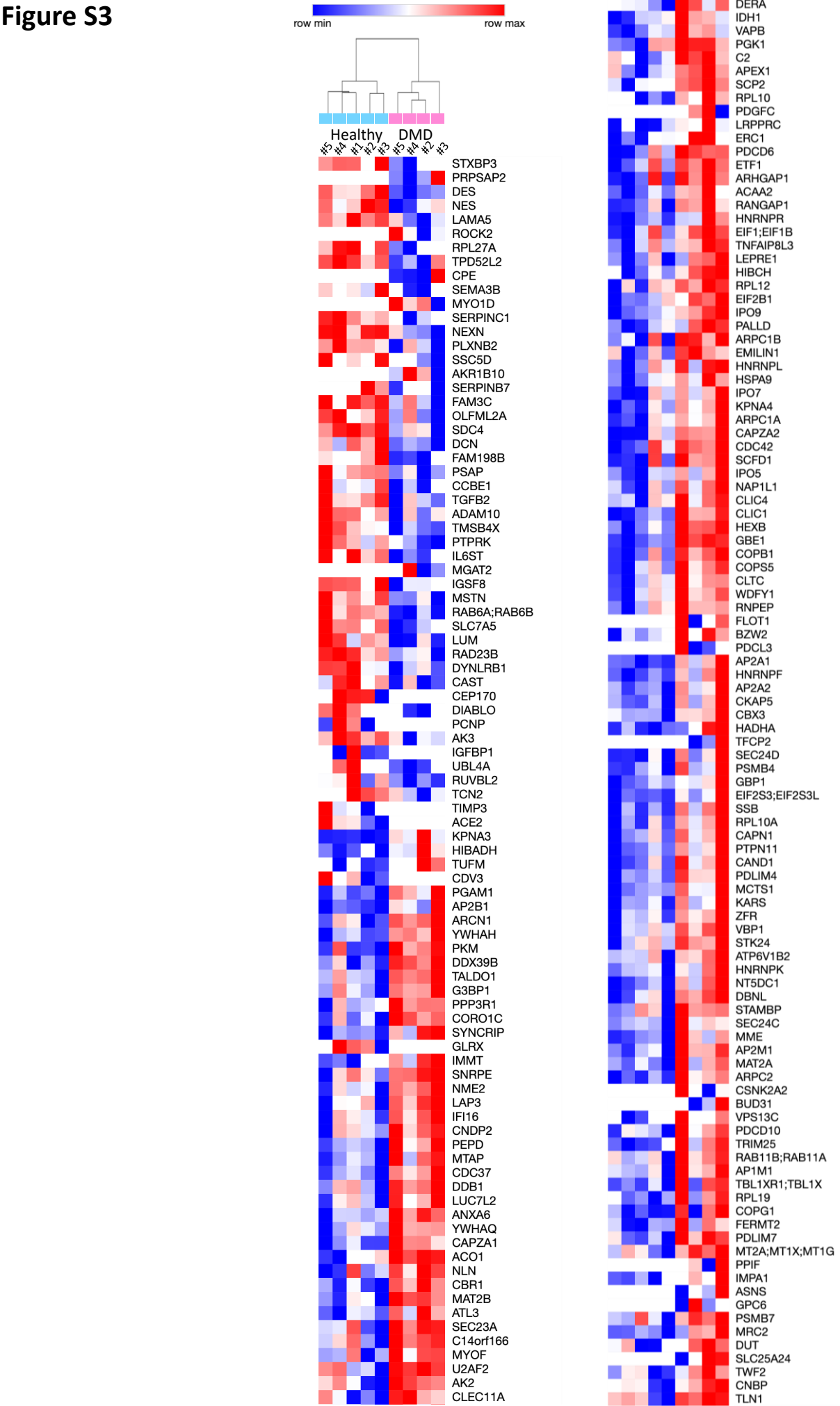

Supplement: Supplementary file 4 — Figure S3 [file 41419_2026_8825_MOESM4_ESM.pdf]

Figure S4

A

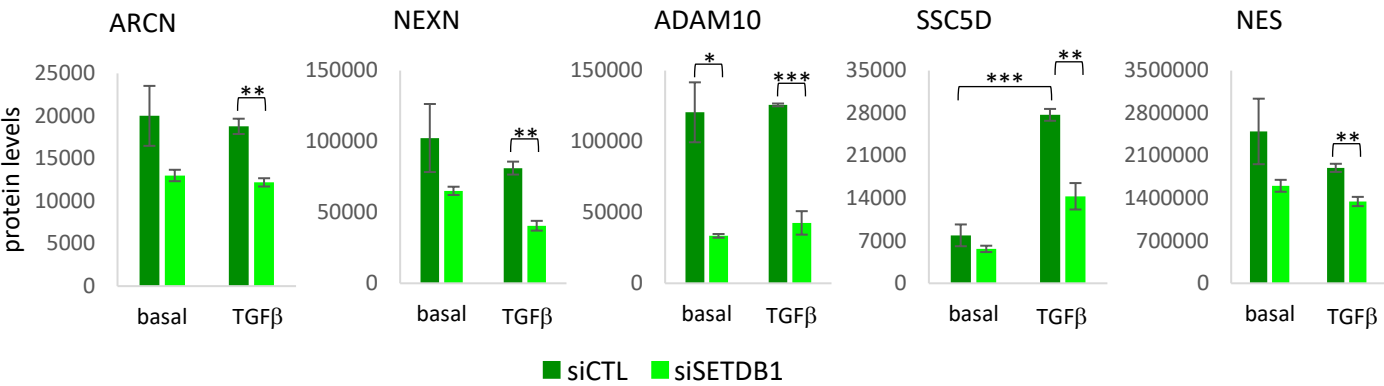

B

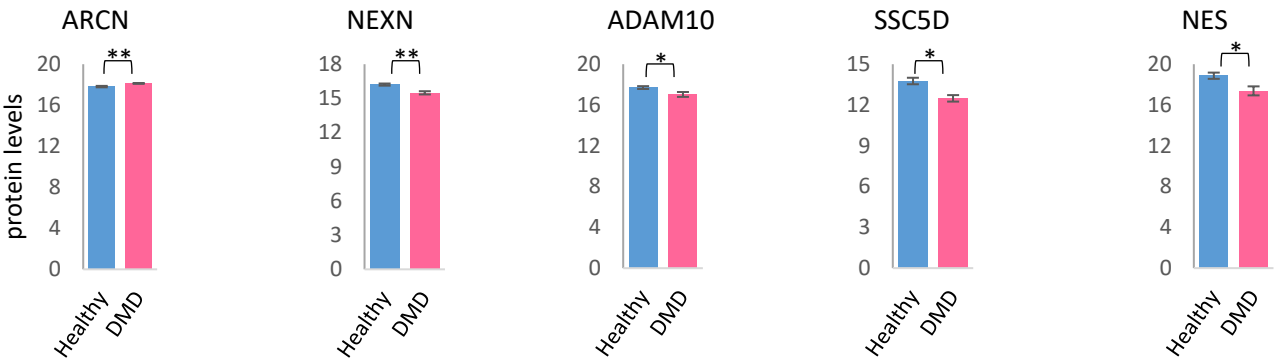

C

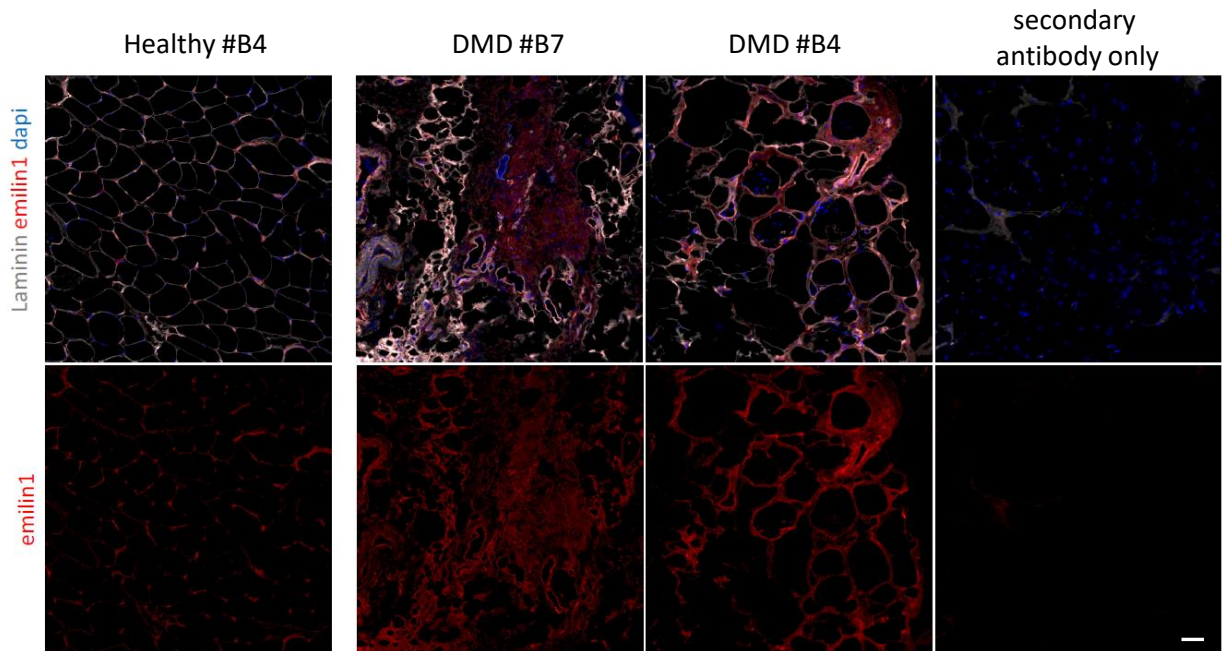

Supplement: Supplementary file 5 — Figure S4 [file 41419_2026_8825_MOESM5_ESM.pdf]

Figure S5

A

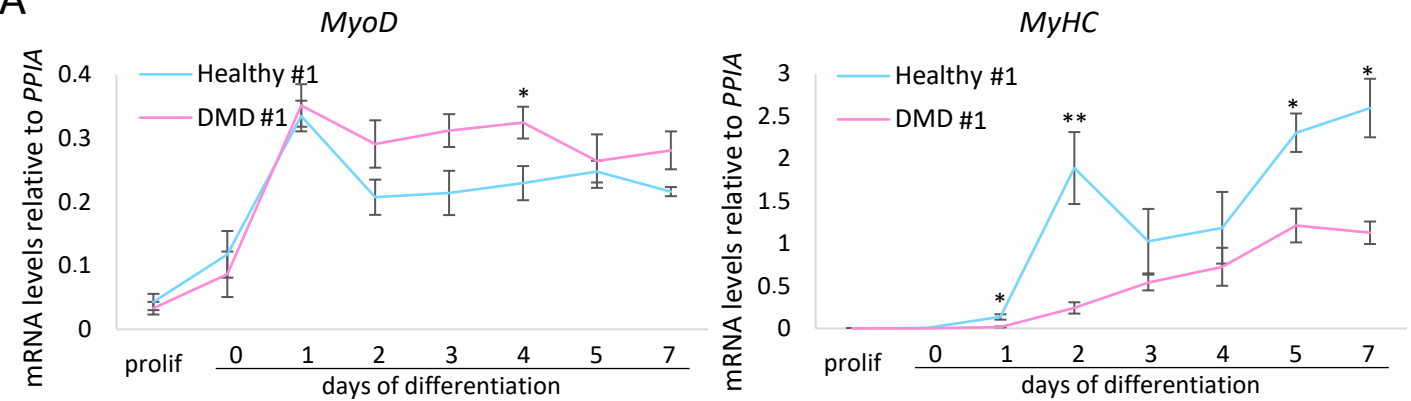

B

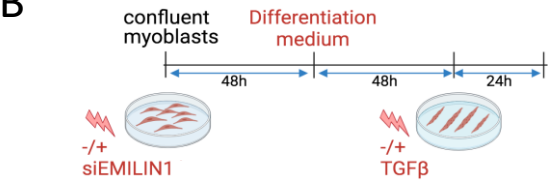

C

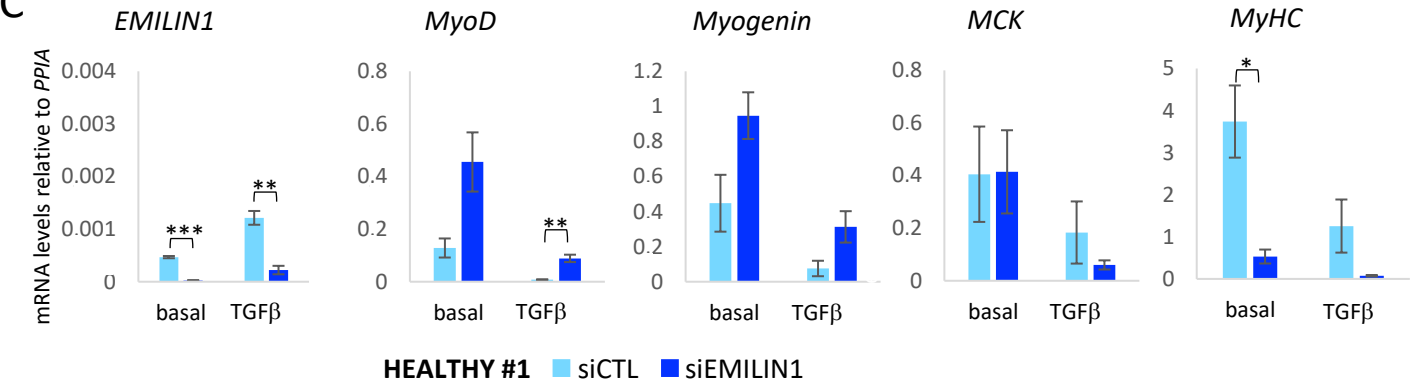

D

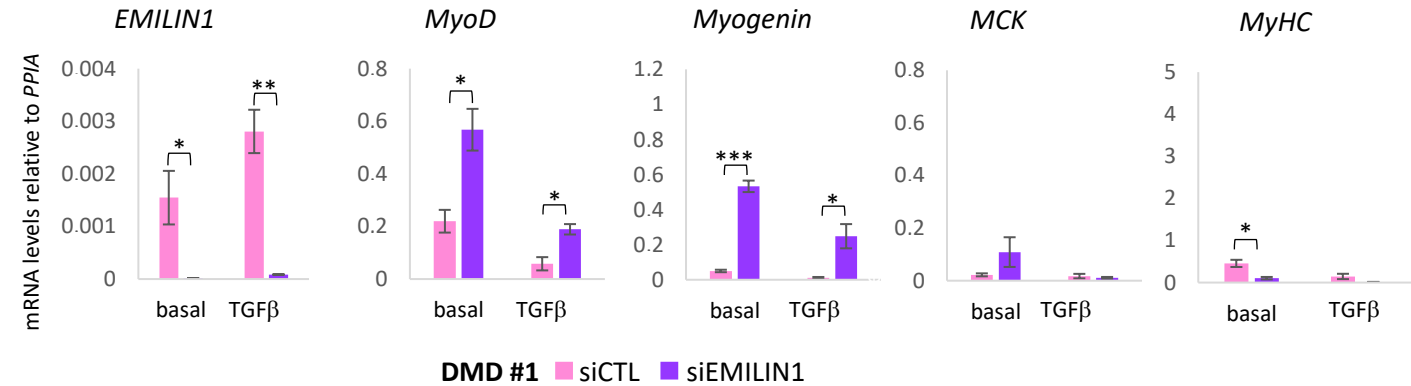

Supplement: Supplementary file 6 — Figure S5 [file 41419_2026_8825_MOESM6_ESM.pdf]

Figure S6

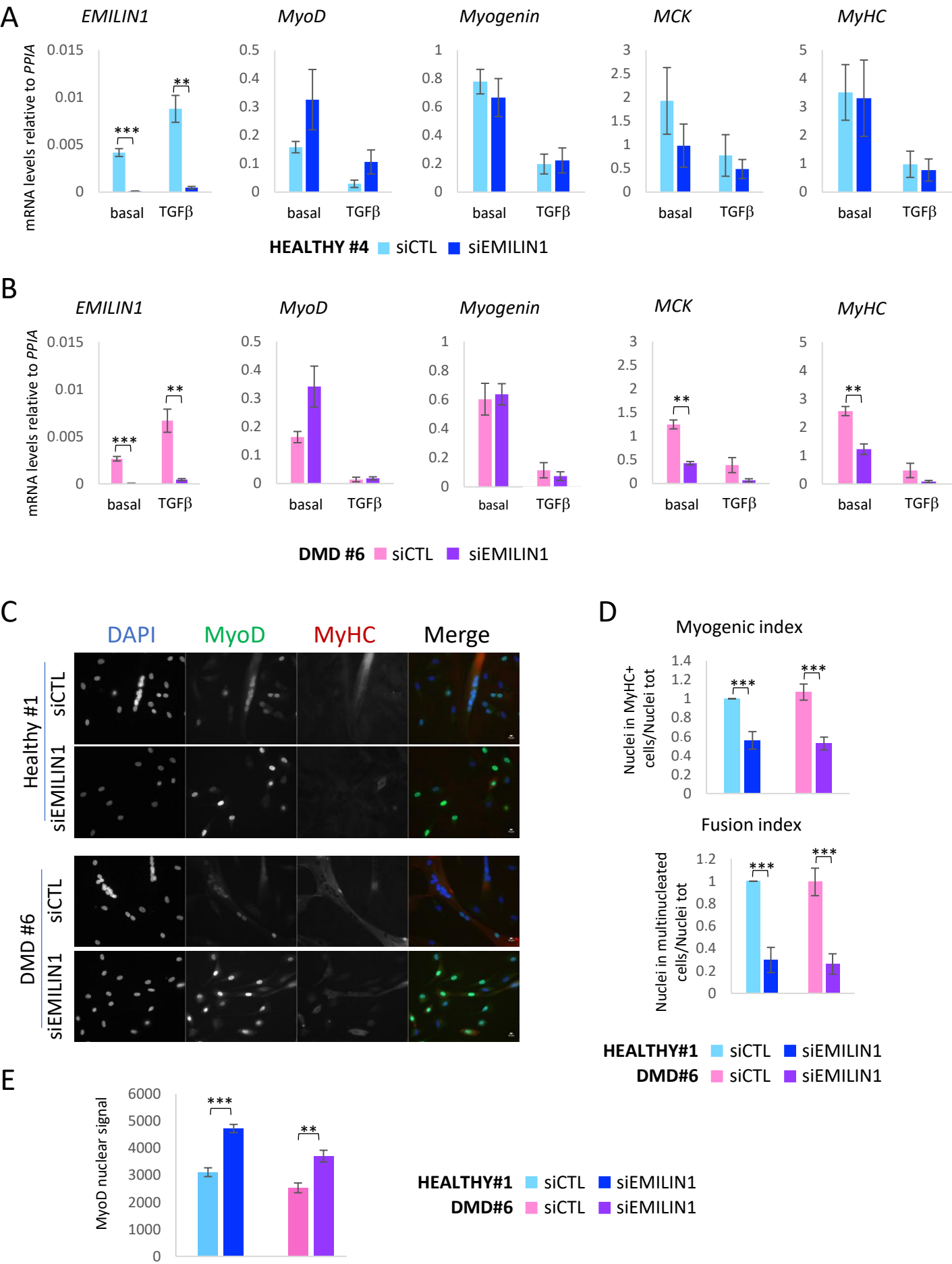

Supplement: Supplementary file 7 — Figure S6 [file 41419_2026_8825_MOESM7_ESM.pdf]

Figure S7

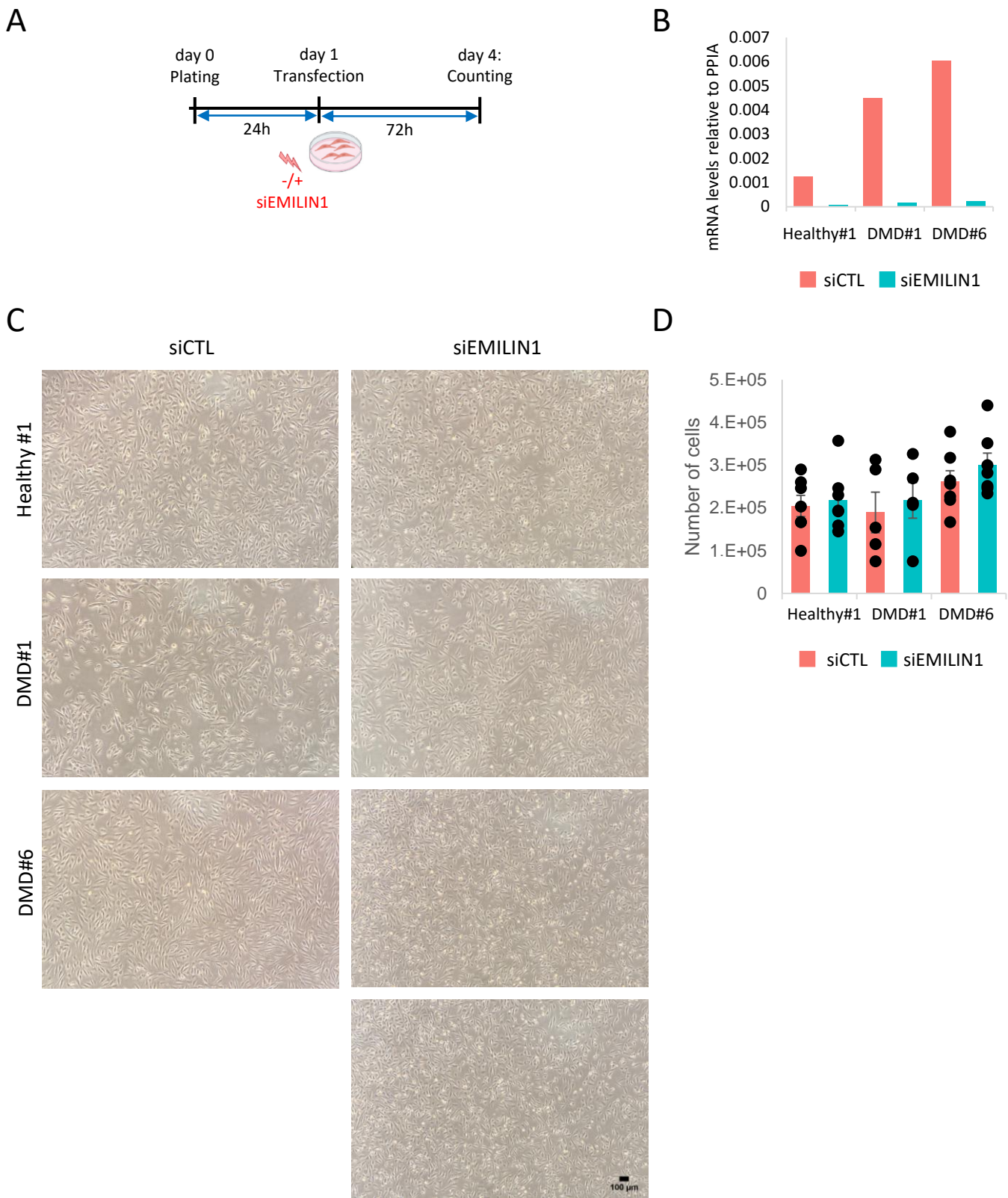

Supplement: Supplementary file 8 — Figure S7 [file 41419_2026_8825_MOESM8_ESM.pdf]

Figure S8

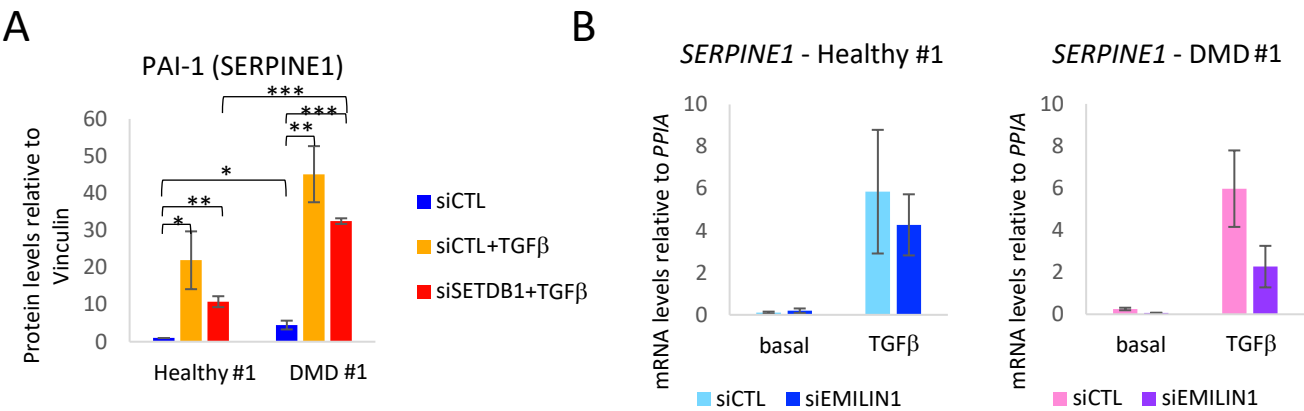

Supplement: Supplementary file 9 — Figure S8 [file 41419_2026_8825_MOESM9_ESM.pdf]
