# Supplementary material for: EMILIN1 emerges as a TGFβ/SETDB1-regulated secreted biomarker in Duchenne muscular dystrophy
Source: Cell Death Dis. 2026 May 9;17(1):611. doi: 10.1038/s41419-026-08825-8 (PMC13323359; doi:10.1038/s41419-026-08825-8)

Supplemental Material : Uncropped western blots

Figure 4G

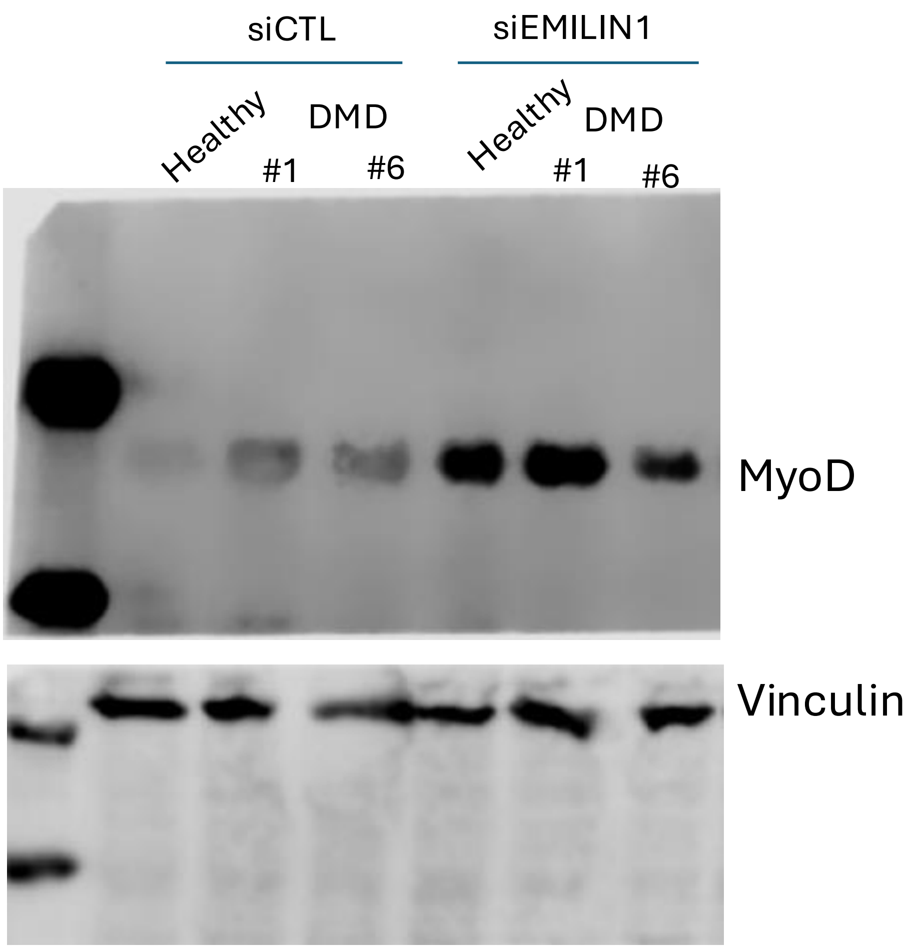

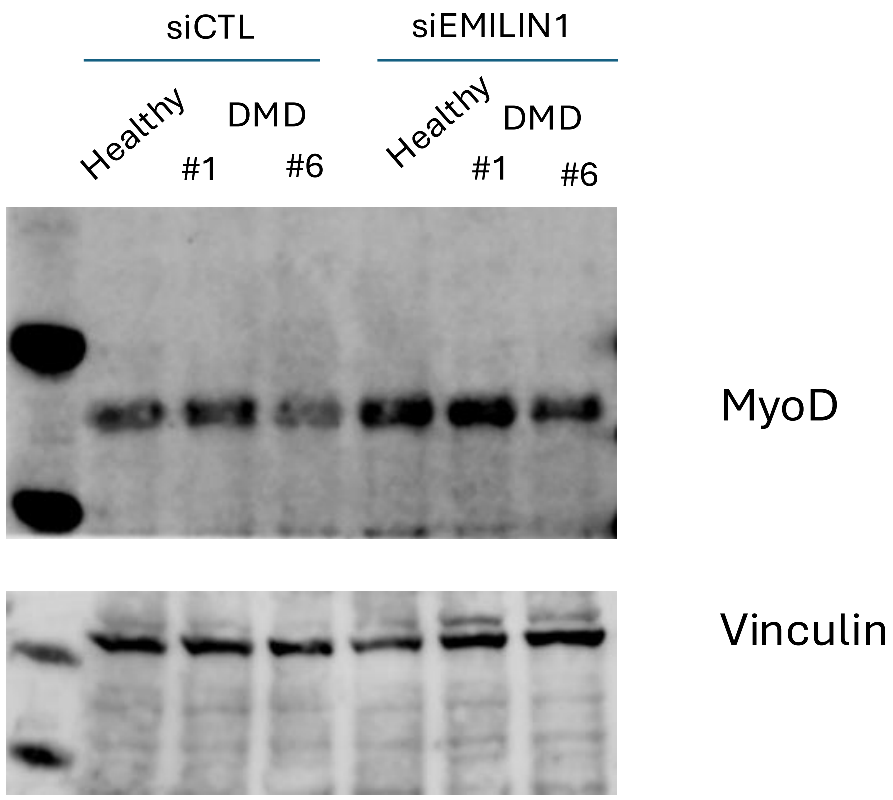

Supplemental Material : Uncropped western blots

Figure 5B

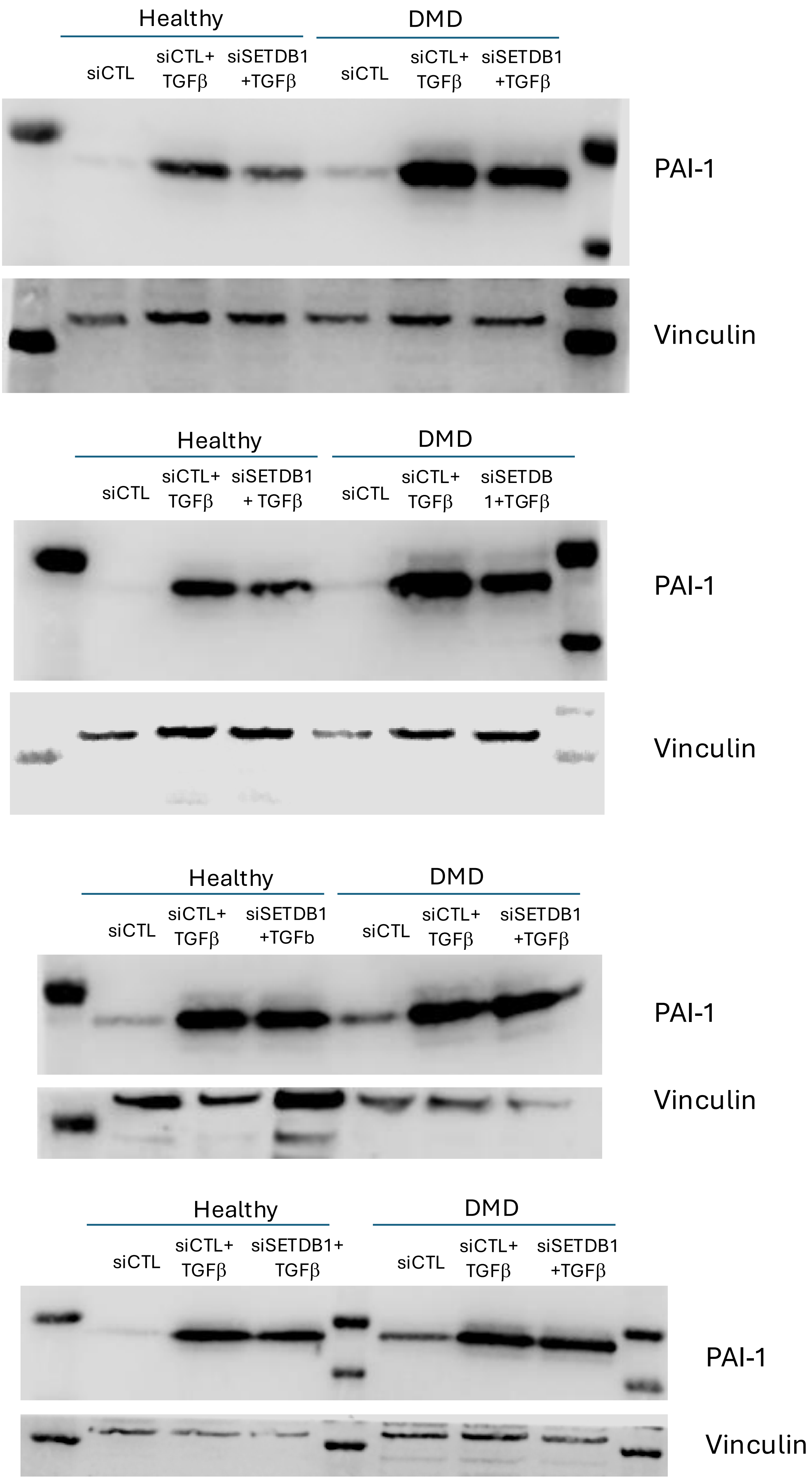

Supplemental Material : Uncropped western blots

Figure 5E

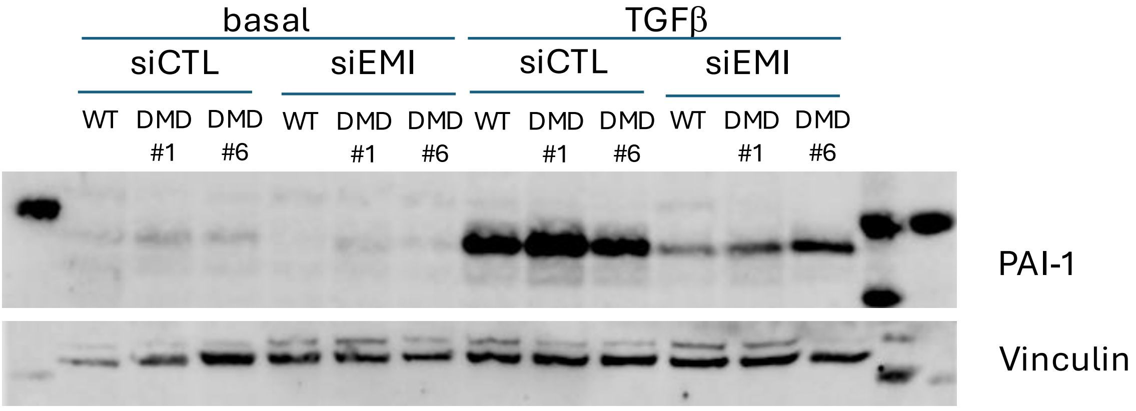

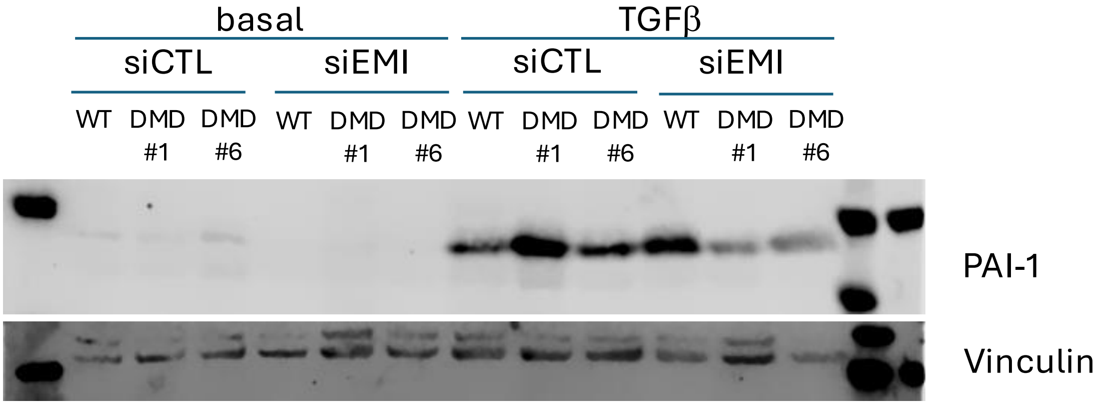

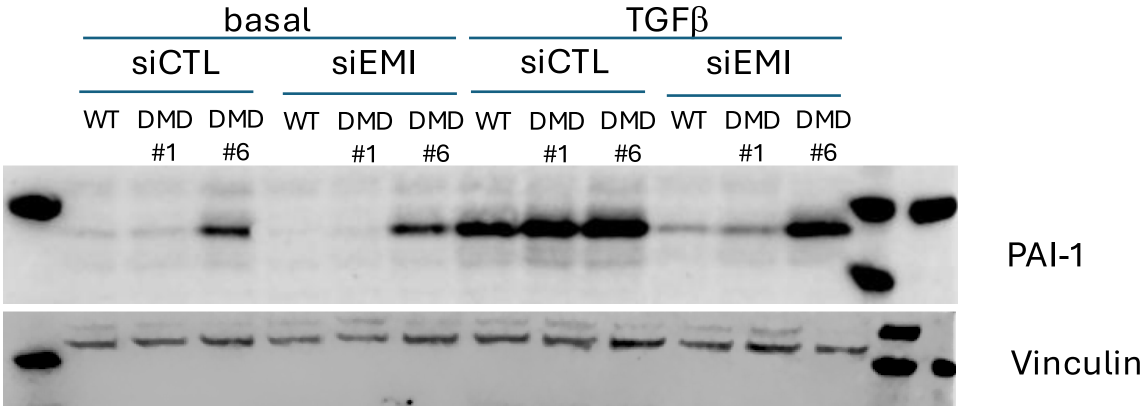

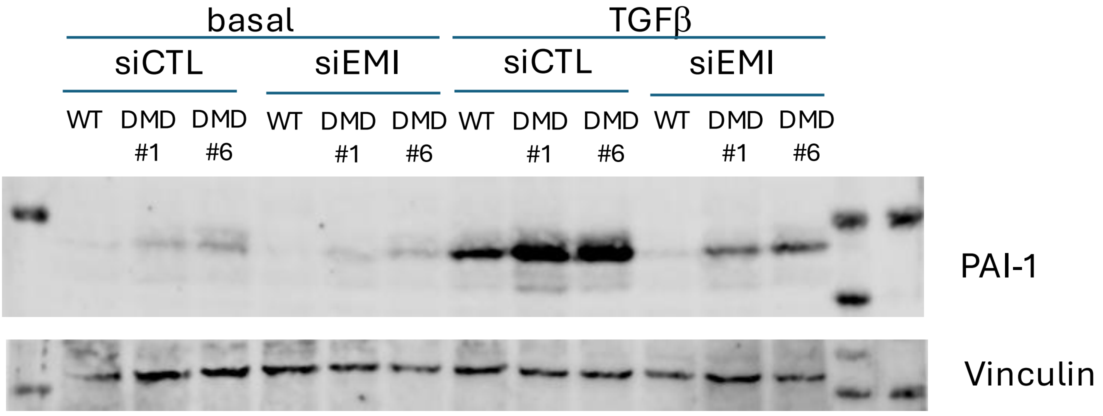

Supplement: Supplementary file 11 — Uncropped western blots [file 41419_2026_8825_MOESM11_ESM.pdf]
